# Quantum-informed machine learning for predicting spatiotemporal chaos with practical quantum advantage

**DOI:** 10.1126/sciadv.aec5049

**Published:** 2026-04-17

**Authors:** Maida Wang, Xiao Xue, Mingyang Gao, Peter V. Coveney

**Affiliations:** ^1^ Centre for Computational Science, University College London, London, UK.; ^2^ Informatics Institute, University of Amsterdam, Netherlands.; ^3^ Centre for Advanced Research Computing, University College London, London, UK.

## Abstract

We introduce a quantum-informed machine learning (QIML) framework for modeling the long-term behavior of high-dimensional chaotic systems. QIML combines a one-time, offline-trained quantum generative model with a classical autoregressive predictor for spatiotemporal field generation. The quantum model learns a quantum prior (Q-Prior) that guides the representation of small-scale interactions and improves the modeling of fine-scale dynamics. We evaluate QIML on the Kuramoto-Sivashinsky equation, two-dimensional Kolmogorov flow, and the three-dimensional turbulent channel flow used as a realistic inflow condition. Across these systems, QIML improves predictive distribution accuracy by up to 17.25% and full-spectrum fidelity by up to 29.36% relative to classical baselines. For turbulent channel inflow, the Q-Prior is trained on a superconducting quantum processor and proves essential: without it, predictions become unstable, whereas QIML produces physically consistent long-term forecasts that outperform leading partial differential equation solvers. Beyond accuracy, QIML offers a memory advantage by compressing multimegabyte datasets into a kilobyte-scale Q-Prior, enabling scalable integration of quantum resources into scientific modeling.

## Teaser

Quantum circuits efficiently learn invariant measures of chaotic systems to stabilize long-term predictions.

## INTRODUCTION

Modeling high-dimensional dynamical systems remains one of the most persistent challenges in computational science. Partial differential equations (PDEs) provide the mathematical backbone for describing a wide range of nonlinear, spatiotemporal processes across scientific and engineering domains (*1*–*3*). However, high-dimensional systems are notoriously sensitive to initial conditions and the floating-point numbers used to compute them (*4*–*7*), making it highly challenging to extract stable, predictive models from data. Modern machine learning (ML) techniques often struggle in this regime: While they may fit short-term trajectories, they fail to learn the invariant statistical properties that govern long-term system behavior. These challenges are compounded in high-dimensional settings, where data are highly nonlinear and contain complex multiscale spatiotemporal correlations.

ML has seen transformative success in domains such as large language models (*8*, *9*), computer vision (*10*, *11*), and weather forecasting (*12*–*15*), and it is increasingly being adopted in scientific disciplines under the umbrella of scientific ML (*16*). In fluid mechanics, in particular, ML has been used to model complex flow phenomena, including wall modeling (*17*, *18*), subgrid-scale turbulence (*19*, *20*), and direct flow field generation (*21*, *22*). Physics-informed neural networks (*23*, *24*) attempt to inject domain knowledge into the learning process, yet even these models struggle with the long-term stability and generalization issues that high-dimensional dynamical systems demand. To address this, generative models such as generative adversarial networks (*25*) and operator-learning architectures such as DeepONet (*26*) and Fourier neural operators (FNO) (*27*) have been proposed. While neural operators offer discretization invariance and strong representational power for PDE-based systems, they still suffer from error accumulation and prediction divergence over long horizons, particularly in turbulent and other chaotic regimes (*28*, *29*). Recent work, such as DySLIM (*30*), enhances stability by leveraging invariant statistical measures. However, these methods depend on estimating such measures from trajectory samples, which can be computationally intensive and inaccurate in all forms of chaotic systems, especially in high-dimensional cases. These limitations have prompted exploration into alternative computational paradigms. Quantum machine learning (QML) has emerged as a possible candidate due to its ability to represent and manipulate high-dimensional probability distributions in Hilbert space (*31*). Quantum circuits can exploit entanglement and interference to express rich, nonlocal statistical dependencies using fewer parameters than their promising counterparts, which makes them well suited for capturing invariant measures in high-dimensional dynamical systems, where long-range correlations and multimodal distributions frequently arise (*32*). QML and quantum-inspired ML have already demonstrated potential in fields such as quantum chemistry (*33*, *34*), combinatorial optimization (*35*, *36*), and generative modeling (*37*, *38*). However, the field is constrained on two fronts: Fully quantum approaches are limited by noisy intermediate-scale quantum (NISQ) hardware noise and scalability (*39*), while quantum-inspired algorithms, being classical simulations, cannot natively leverage crucial quantum effects such as entanglement to efficiently represent the complex, nonlocal correlations found in such systems. These challenges limit the standalone utility of QML in scientific applications today. Instead, hybrid quantum-classical models provide a promising compromise, where quantum submodules work together with classical learning pipelines to improve expressivity, data efficiency, and physical fidelity. In quantum chemistry, this hybrid paradigm has proven feasible, notably through quantum mechanical/molecular mechanical coupling (*40*, *41*), where classical force fields are augmented with quantum corrections. Within such frameworks, techniques such as quantum-selected configuration interaction (*42*) have been used to enhance accuracy while keeping the quantum resource requirements tractable. In the broader landscape of quantum computational fluid dynamics, progress has been made toward developing full quantum solvers for nonlinear PDEs. Recent works by Liu *et al.* (*43*) and Sanavio *et al.* (*44*, *45*) have successfully applied Carleman linearization to the lattice Boltzmann equation, offering a promising pathway for simulating fluid flows at moderate Reynolds numbers. These approaches, typically using algorithms such as Harrow-Hassidim-Lloyd (HHL) (*46*), promise exponential speedups but generally necessitate deep circuits and fault-tolerant hardware.

Quantum-enhanced machine learning (QEML) combines the representational richness of quantum models with the scalability of classical learning. By leveraging uniquely quantum properties such as superposition and entanglement, QEML can explore richer feature spaces and capture complex correlations that are challenging for purely classical models. Recent successes in quantum-enhanced drug discovery (*37*), where hybrid quantum-classical generative models have produced experimentally validated candidates rivaling state-of-the-art classical methods, demonstrate the practical potential of QEML even before full quantum advantage is achieved. Despite these strengths, practical barriers remain. QEML pipelines require repeated quantum-classical communication during training and rely on costly quantum data-embedding and measurement steps, which slow computation and limit accessibility across research institutions. Moreover, most current QEML applications focus on microscopic problems, such as atomic and molecular systems in quantum chemistry, with little exploration of macroscopic phenomena such as nonlinear PDEs and turbulent flows, where long-term stability and statistical fidelity pose distinct challenges (*47*). In parallel, the pursuit of quantum advantage in pure quantum computing faces closely related challenges. While theoretical speedups are well established for specific algorithms, identifying practical, application-level advantages remains difficult in realistic settings. This difficulty is exacerbated by the overhead associated with data encoding (*31*), the impact of noise in near-term devices (*39*), and fundamental limits on information extraction imposed by Holevo’s bound (*48*). As a result, many demonstrations of quantum advantage to date rely on analog sampling tasks (*49*), quantum-native data, or postprocessing of quantum outputs (*50*) rather than real-world, classically relevant problems.

**Fig. 1.**
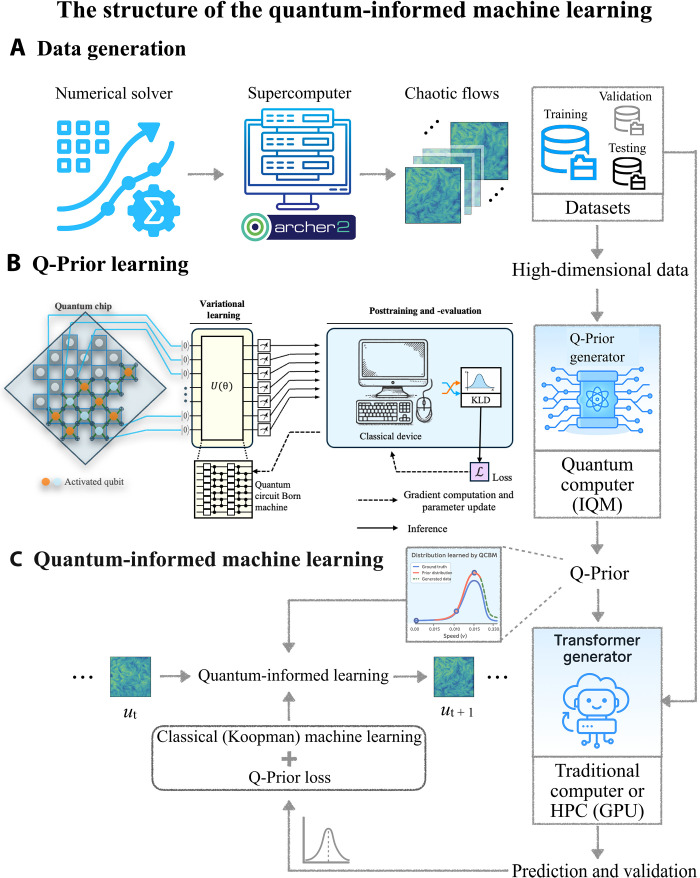
Architecture of the QIML framework. (**A**) High-dimensional dynamical flow fields are generated using high-resolution numerical solvers and used to construct training, validation, and test datasets. (**B**) A quantum circuit is trained on superconducting hardware to learn invariant statistical properties from the data (Q-Prior). (**C**) The learned Q-Prior is integrated into a classical high-performance computer (HPC) cluster, guiding it toward physically consistent predictions and improved long-term stability.

To address these limitations, we propose a quantum-informed machine learning (QIML) architecture, as shown in Fig. 1M, a specialized hybrid quantum-classical framework for chaotic systems. The quantum component is a sample-based quantum generator grounded on the quantum circuit Born machine (*51*, *52*), trained once offline on quantum hardware to learn invariant statistical properties directly from observational data. The resulting compact quantum prior (Q-Prior) can be deployed broadly and efficiently without ongoing quantum-classical iteration, eliminating the need for repeated access to quantum hardware during training. This design is specifically tailored to learning the dynamics of nonlinear PDEs. These properties capture complex, high-dimensional distributions compactly by leveraging entangled quantum states. Unlike standard deep learning surrogates, such as fully connected or convolutional networks that require large parameter counts to represent such structures, the quantum generators operate in exponentially large Hilbert spaces, enabling expressive modeling with as few as 10 to 15 qubits for the systems here. Crucially, this framework circumvents the data-loading bottleneck common to many QML algorithms. The generator does not need to encode raw data onto quantum states; instead, it learns a compressed, generative model of the data’s underlying statistical properties. The model is trained with a sample-based maximum mean discrepancy (MMD) loss (*53*), enabling it to learn the quantum representation without knowing the data distribution. After the training of the quantum generator, the resulting Q-Prior is then integrated into an autoregressive, unitary-enhanced Koopman-based ML framework (*54*). This architecture is powerful for learning dynamical systems, as it can linearize nonlinear dynamics in a lifted observable space, making it effective for capturing coherent structures (*55*, *56*). However, for high-dimensional chaotic systems, its ability to maintain predictive stability remains a notable challenge, often failing to preserve the systems’ physical invariant measures over extended rollouts (i.e., recursive 1-in-1-out predictions where each forecasted state is fed back as input for the next step). This is particularly true when modeling data for which no closed-form governing equation is known—as is the case for a two-dimensional (2D) cross section extracted from a 3D turbulent domain. We address this limitation by embedding the Q-Prior as a differentiable constraint directly within the loss function. This composite loss, which penalizes divergence from the Q-Prior, imposes a powerful statistical regularization on the evolution of the Koopman operator, guiding it to learn a physically consistent and stable long-term dynamic. We evaluate our QIML framework against its classical counterpart without Q-informed guidance, as well as two leading ML models: the FNO for general PDE-based systems and the Markov neural operator (MNO) (*57*) specifically designed for chaotic dynamics. Our QIML framework outperforms all baseline models while requiring orders-of-magnitude fewer trainable parameters.

## RESULTS

### Numerical set up

The selected test cases span increasing levels of physical and dynamical complexity. We begin with the Kuramoto-Sivashinsky (KS) equation, a PDE exhibiting spatiotemporal chaos. The solution is discretized into 512 spatial grid points, resulting in a 512D state vector at each time step. Next, we consider 2D Kolmogorov flow on a 64 × 64 spatial grid (dimension = 4096), governed by the 2D incompressible Navier-Stokes equations in a periodic domain, where the quantum generator is trained in emulation to extract turbulent invariant measures. Last, we examine cross sections of a 3D turbulent channel flow (TCF), simulated using the lattice Boltzmann method (LBM), which is numerically equivalent to solving the Navier-Stokes equations (*58*). For the training process, the raw cross sections are downsampled and discretized onto a 64 × 64 spatial grid, resulting in a 4096D state vector. The Q-Prior for TCF is trained on superconducting quantum processors. While the full flow field is simulated numerically, the extracted 2D cross-sectional datasets used in our analysis do not satisfy any closed-form governing equation. This distinction highlights a further step in complexity: moving from lower-dimensional systems with explicit dynamics to high-dimensional observational data without accessible evolution laws, thereby testing the capacity of QIML to generalize across different scenarios. Throughout this study, “ground truth” or “raw data” refers to the high-resolution simulation data generated by established numerical solvers. While this raw simulation data have a large memory footprint, the quantum-informed priors offer parameter efficiency and memory advantage. They capture the flow’s essential statistical features using fewer than 300 trainable parameters, occupying only a few megabytes of storage, which corresponds to a data storage reduction of over two orders of magnitude compared to the raw data. This approach enables a substantial reduction in data storage requirements compared to using the raw simulation data (see section Materials and Methods and Supplementary sections S2 and S3). The classical components of the models were trained on a single NVIDIA A100 80 GB GPU using default settings with 32-bit floating-point precision (for details, see section S4).

### KS equation

We first apply our QIML framework to learn the solution of the KS equation, which is given by

$$\frac{\partial u}{\partial t}+u\frac{\partial u}{\partial x}+\nu \frac{\partial^{2}u}{\partial x^{2}}+\mu \frac{\partial^{4}u}{\partial x^{4}}=0$$ (1)

where u(x,t) denotes a scalar field representing the state of the system, with u:[0,L]×[0,∞)→ℝ, x∈[0,L] representing the spatial domain and t∈[0,∞) the time; ν and μ are constants which are set to 1 for this study. The spatial domain is discretized using *N* = 512 equidistant grid points. Raw data are generated as described in section S3. The dataset consists of 1200 trajectories, where each trajectory is made up of 2000 time frames. The spatial domain for each frame is discretized using 512 grid points. We partition the trajectories chronologically into 80% training, 10% validation, and 10% test sets. For this system, both the classical Koopman ML (without Q-Prior) and the main QIML model (with Q-Prior) are trained for 500 epochs. The Q-Prior for the QIML configuration is generated by a sample-based quantum generator using 10 qubits (2^10^ = 1024 basis states) and 120 trainable parameters. Full architectural and training details for both configurations are provided in section S7.

**Fig. 2.**
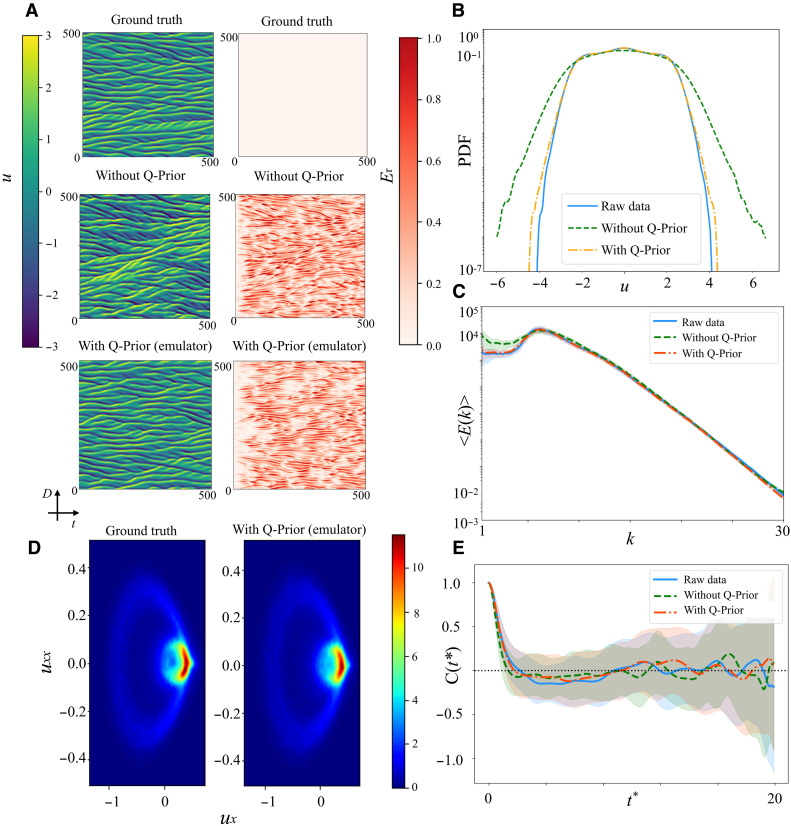
Evaluation of the QIML framework on the KS system. (**A**) This panel displays time-averaged mean velocity fields (left column) and corresponding relative error *E*_r_ maps (right column) across the ground truth, the classical ML without Q-Prior, and the quantum-informed ML model with Q-Prior (from top to bottom). (**B**) This panel presents the probability distribution of *u*. (**C**) This panel shows the energy spectrum 〈E(k)〉 as a function of spatial wave number *k*, characterizing kinetic energy distribution across spectral modes. (**D**) This panel visualizes the invariant density when dynamics are projected into the $\left(u_{x},u_{\mathrm{xx}}\right)$ space, revealing the geometric support of the underlying invariant measure. (**E**) This panel plots the temporal autocorrelation, denoted by $C\left(t^{*}\right)$, computed from the time series of the field *u* averaged over all spatial points. The correlation is shown as a function of the dimensionless time lag $t^{*}=t/t_{\text{Lyapunov}}$.

Figure 2 presents the QIML evaluation results on the KS system, comparing two configurations against the numerical simulation: a classical ML model trained without Q-Priors (without Q-Prior) and a QIML model incorporating a prior trained in simulation (with Q-Prior). Both ML models perform inference in an autoregressive manner: Starting from an initial condition at time step *t*_0_ = 0, the model predicts the next frame at each subsequent step and recursively continues this process up to *t*_end_ = 500 time steps to ensure long-term statistics. Figure 2A shows the *u* evolution alone the time: with 512 initial states and 500 prediction timesteps, and the corresponding relative error *E*_r_ evolution. The error metric definition is presented in section S1. From top to bottom, each row corresponds to the ground truth, the ML model without Q-Prior configuration, and the QIML model with Q-Prior configuration. These subpanels are computed over the test dataset to visualize long-term statistical characteristics of the predictions. In the “without Q-Prior” configuration, the model reproduces the general structure of the ground truth. However, over time, the predicted stripe patterns drift upward, revealing noticeable discrepancies in the associated error map. This indicates that the model accumulates errors during long-term prediction, likely due to the absence of prior information to guide the dynamics. In contrast, the “with Q-Prior” model demonstrates much closer alignment with the ground truth. The predicted fields preserve fine-scale features and capture the parallel stripe structures effectively. In additioon, the corresponding error maps exhibit substantially lower and more spatially diffuse errors. These results suggest that incorporating the Q-Prior enhances the model’s stability and accuracy in long-term forecasting. Figure 2B presents the probability density function (PDF) of *u* for each configuration. The distribution is obtained via the entire spatiotemporal test domain (512 states in space and 500 prediction steps in time), allowing for a comparison of global statistical fidelity. The raw test data (solid blue line) serve as the ground truth. The model without Q-Prior (green dashed line) deviates from the raw data, particularly in the tails of the distribution, where it tends to overestimate the probability. In contrast, the model with Q-Prior (orange dash-dotted line) closely matches the ground truth across the full velocity range, especially in the tails. This improved statistical fidelity is confirmed by a quantitative analysis of the overall prediction error; the inclusion of the Q-Prior results in a 17.25 ± 5.25% reduction in the mean squared error (MSE) of the predicted fields with respect to the ground truth over the first 100 steps. This effect becomes even more pronounced in the distribution tails (events where ∣u∣>4), where the error is suppressed by nearly two orders of magnitude. Moreover, an analysis of the spectrum demonstrates superior performance across all energy scales, achieving a 29.36 ± 9.01% reduction in MSE for this metric. This demonstrates that incorporating the Q-Prior allows the ML model to more accurately reproduce the statistical characteristics of the system, including rare events. Figure 2C shows the energy spectrum 〈E(k)〉 as a function of the wave number *k*, comparing the raw data to the ML model with and without Q-Prior. The energy spectrum of the raw data follows a smooth decay with increasing *k*, reflecting the distribution of energy across spatial frequencies. The model without Q-Prior overestimates the energy in the large scale, deviating from the ground truth. However, the model with Q-Prior closely aligns with the raw data across all wave numbers, capturing both large-scale and small-scale (high-*k*) behavior of the system. To further visualize the invariant structures underlying the KS system, we project both the ground truth and the Q-Prior configuration into the $\left(u_{x},u_{\mathrm{xx}}\right)$ phase space, as shown in Fig. 2D. The resulting density plots depict the support of the system’s invariant measure, providing insight into its long-term statistical behavior. Over an extended prediction, the model with Q-Prior closely aligns with the ground truth in phase space, accurately capturing the geometry and concentration of the invariant set. This qualitative agreement highlights the Q-Prior model’s ability to preserve the system’s underlying statistical structures over time. Figure 2E illustrates the temporal autocorrelation $C\left(t^{*}\right)$ of the velocity field, averaged over all spatial points, as a function of the dimensionless time lag $t^{*}=t/t_{\text{Lyapunov}}$. Autocorrelation measures how strongly the system retains correlation with its past states over time. Specifically, its calculation is presented in section S1. In this plot, all cases exhibit a decay in autocorrelation, eventually showing no correlation with the initial state. The configuration with Q-Prior demonstrates stronger short-term correlation (the same as the ground truth data) compared to the model without Q-Prior, indicating better preservation of temporal dynamics in the early prediction period. These outcomes demonstrate that our designed Q-Prior can regularize the classical ML model, preserving both long-scale and small-scale structures and improving long-term statistical accuracy.

### 2D Kolmogorov flow

Next, we apply our QIML framework to learn the dynamics of the 2D Kolmogorov flow, a canonical example of incompressible Navier-Stokes dynamics under sinusoidal forcing. The governing equations are

$$\frac{\partial u}{\partial t}+\left(u\cdot \nabla \right)u=-\nabla p+\nu \nabla^{2}u+F$$ (2)

∇⋅u=0 (3)

where **u** is the velocity field and *p* represents the pressure. Here, **u** denotes the velocity field on the 2D cases. In the algorithmic expressions, we revert to the simpler symbol *u*, using it as a generic placeholder for both 1D scalar and 2D velocities, because the learning procedure applies uniformly across dimensionalities. ν is the kinematic viscosity, and the forcing term is given by F(x,y)=[Fsin(ky),0] for some amplitude *F* and wave number *k*.

The domain is periodic in both *x* and *y* directions: (x,y)∈$\left[0,L_{x}\right]\times \left[0,L_{y}\right]$ with *L_x_* = *L_y_* = 64 grids. The data source is presented in section S4. We use 80% for training, 10% for validation, and 10% for testing. For this system, both the classical Koopman ML (without Q-Prior) and the main QIML model (with Q-Prior) are trained for 500 epochs. For QIML, the quantum generator uses *n* = 10 qubits (2^10^ = 1024 basis states) and 180 trainable parameters. Detailed network architectures, training hyperparameters, and data generation procedures are provided in section S7.

**Fig. 3.**
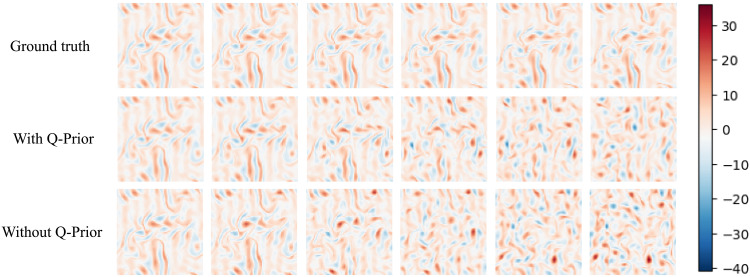
Early-time chaotic evolution of streamwise velocity fields. The streamwise velocity rollout of ground truth (top), the QIML with Q-Prior (middle), and the classical model without Q-Prior (bottom) given the same initial state at $\hat{t}=0.0,0.2,0.4,0.6,0.8,1.0$ (from left to right), corresponding to Lyapunov times $t^{*}=0.0,0.04,0.08,0.12,0.16$, and 0.20, respectively.

**Fig. 4.**
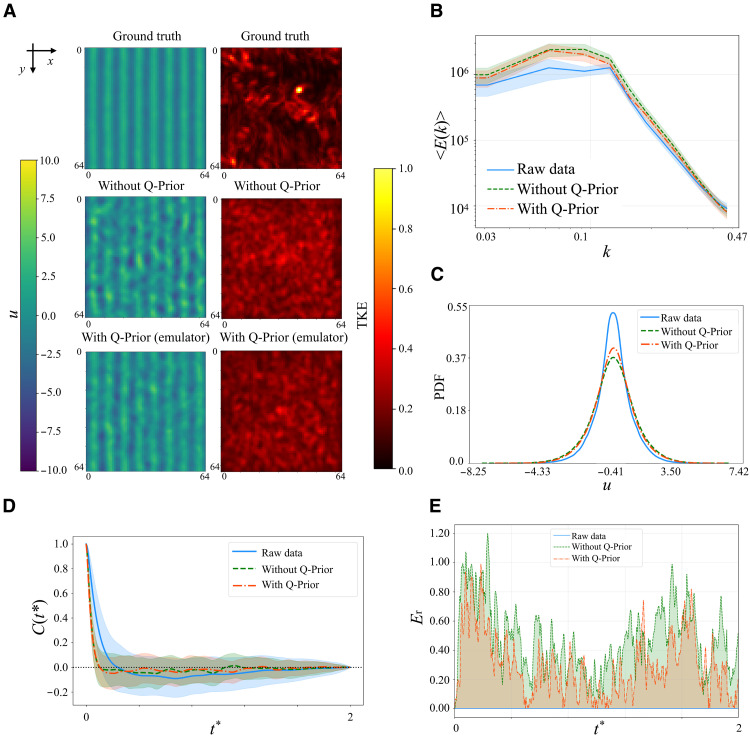
Evaluation of the QIML framework on the 2D Kolmogorov flow. (**A**) This panel displays time-averaged mean *u* fields (left column) and corresponding TKE fields (right column) for the ground truth, the classical model without Q-Prior, and the quantum-informed model with Q-Prior. These maps are evaluated on the test set, highlighting long-term flow patterns and energy distribution. (**B**) This panel presents the ensemble-averaged energy spectrum 〈E(k)〉 as a function of spatial wave number *k*. (**C**) This panel shows the PDF of the field variable *u*, comparing the distribution from the raw test data with the PDFs predicted by the models with and without the Q-Prior. (**D**) This panel plots the temporal autocorrelation $C\left(t^{*}\right)$ of the velocity field as a function of dimensionless time $t^{*}$. (**E**) This panel reports the normalized relative error, *E*_r_, with respect to the ground truth, calculated over time on the same validation trajectory for a 1000-step rollout.

Figure 3 presents the predicted streamwise velocity fields over a 20-step autoregressive rollout ($t_{\text{window}}=20$), where the time is normalized as $\hat{t}=t/t_{\text{window}}$. The snapshots qualitatively demonstrate that the QIML model preserves the coherent vortical structures of the ground truth with higher fidelity and for a longer duration compared to its classical counterpart, in which these features decay more rapidly into a disorganized state. Figure 4 reports the comparative performance of the baseline ML model (without Q-Prior) and the quantum-informed ML model (with Q-Prior) on the 2D Kolmogorov flow dataset. Figure 4A shows the time-averaged velocity field and the corresponding turbulent kinetic energy (TKE) field across three configurations: the ground truth from simulation, the classical model trained without Q-Prior, and the quantum-informed model trained with Q-Prior. All models are evaluated on the same test data. Both the with Q-Prior and without Q-Prior models perform autoregressive (one frame in, one frame out) inference over a horizon of 1000 which is around 2 Lyapunov times $t_{\text{Lyapunov}}=500$ (*59*). Beyond *O*(1) Lyapunov time, trajectory-wise prediction is not expected; therefore we assess long-horizon performance via statistical fidelity. The visualizations span a 2D domain of size 64 × 64 in the *xy* plane. The time-averaged velocity field is plotted in the left column, and the corresponding TKE field is shown in the right column. The ground truth velocity field (top left) exhibits coherent, stripe-like structures, and its TKE field (top right) reveals concentrated regions of high energy, indicating the presence of organized flow features. The model without Q-Prior (middle row) shows degraded spatial coherence in the velocity field, with disrupted patterns and more noise-like structures. In contrast, the model with Q-Prior (bottom row) recovers a more organized velocity field, better preserving the stripe patterns observed in the ground truth. Its associated TKE field also aligns more closely with the ground truth. Figure 4B presents the ensemble-averaged energy spectrum 〈E(k)〉 as a function of the wave number *k* for the raw data (solid blue line), the ML model without Q-Prior (green dashed line), and the model with Q-Prior (orange dash-dotted line). The spectrum from the model without Q-Prior overestimates the energy spectrum at low and intermediate wave numbers. However, the Q-Prior aligns closer to the raw data across the whole spectrum. Then, we further examine the overall velocity distributions. As illustrated in Fig. 4C, we compare the predicted velocity PDFs from both ML models. The raw data exhibit a sharply peaked distribution with heavy tails, characteristic of the system’s inherent velocity fluctuations. Both ML models capture the general symmetric shape of the distribution but tend to slightly underestimate the peak and overestimate the spread. Among them, the model with Q-Prior more closely matches the raw data, particularly around the peak and in the tails, indicating improved accuracy in reproducing the system’s statistical behavior. This is quantitatively supported by a 6.57 ± 3.68% reduction in the MSE for the Q-Prior model’s predictions compared to the classical model without the prior over the first 100 steps. This advantage is particularly pronounced in the high-probability peak of the distribution (where ∣u∣<2), where the MSE reduction for the Q-Prior model increases to 10.39 ± 4.23%. On a global scale, this superior performance is also reflected in the energy spectrum, where the Q-Prior achieves a 14.16% MSE reduction. These results further support the conclusion that incorporating the Q-Prior enhances the ML model’s ability to recover velocity statistics. We also examined the autocorrelation of the field over time. Figure 4D plots the autocorrelation $C\left(t^{*}\right)$ of the velocity field as a function of dimensionless time $t^{*}=t/t_{\text{Lyapunov}}$. The correlation is computed over 100 randomly sampled locations, with shaded regions indicating one SD. Both models reproduce long-term decay in autocorrelation, followed by fluctuations around zero, consistent with the expected loss of memory in a chaotic or turbulent system. To further analyze the model, Fig. 4E reports the normalized relative autocorrelation error *E*_r_ with respect to the ground truth. The raw data serve as the reference baseline with zero error. Across the entire temporal range, the QIML model incorporating the Q-Prior consistently exhibits lower autocorrelation error than the classical model trained without the Q-Prior. These observations confirm that the quantum-informed Q-Prior can provide additional information that improves classical ML fidelity and velocity statistics and enhances long-horizon accuracy.

### TCF inflow generation

**Fig. 5.**
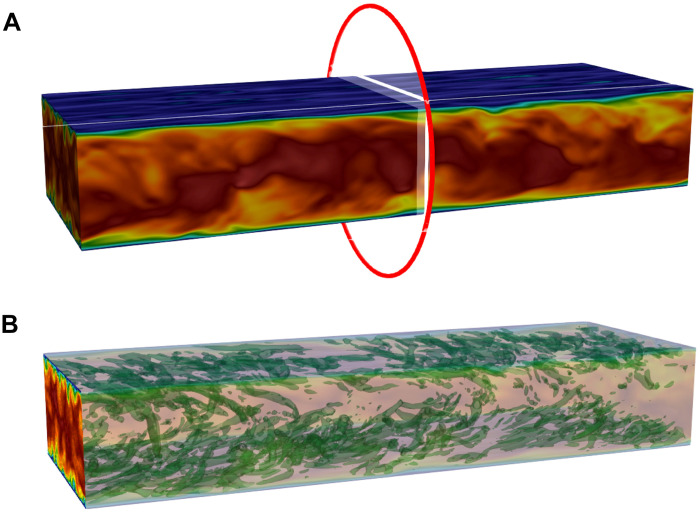
Generation of synthetic turbulent inflow from TCF data. (**A**) Instantaneous flow field from a TCF simulation, representing the raw data used for turbulent inflow generation. The red ellipse indicates the cross section where velocity field data are extracted. (**B**) The QIML generative model can be used as turbulent inflow conditions in 3D TCF for LESs.

We further evaluate our QIML framework on TCF, a representative case of wall-bounded turbulence. In this setting, the training data are obtained from the midplane cross section of a fully developed periodic TCF at friction Reynolds number *Re*_τ_ = 180. The reference data have been validated with the direct numerical simulation (DNS) data (see section S5) (*60*). The reference data are generated using the LBM solver, which serves as an efficient alternative to directly solving the Navier-Stokes equations (*58*, *61*). A large eddy simulation (LES) formulation is used to capture the large-scale turbulent structures (*18*, *62*). Figure 5A shows the instantaneous velocity field extracted from the high-fidelity TCF simulation, with the red ellipse marking the cross section used as the source data for inflow synthesis. The governing equations used for generating the data are provided in section S5. The detailed description of the data generation setup is in section S8. The channel flow snapshots are split 80:10:10 into training, validation, and test sets. For this system, both the classical Koopman ML model (without Q-Prior) and the main QIML model (with Q-Prior) are trained for 500 epochs. For QIML, we use a 15-qubit quantum generator (2^15^ = 32,768 basis states) with 240 trainable parameters. Detailed network architectures, training hyperparameters, and data generation procedures are provided in section S7. Figure 5B depicts the corresponding synthetic turbulent inflow generated by the QIML model, demonstrating its capability to produce physically consistent inflow conditions for large-eddy simulations (*62*).

**Fig. 6.**
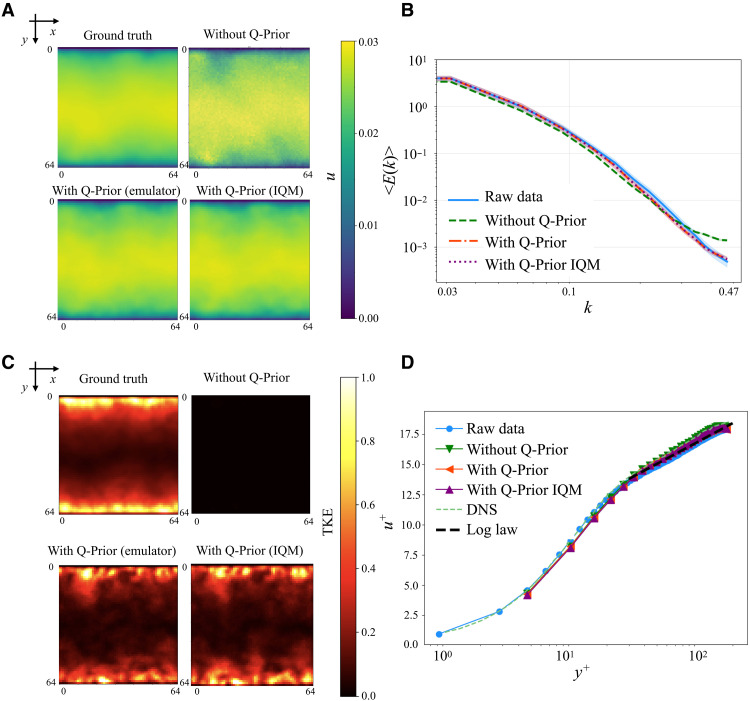
Evaluation of the QIML framework on the TCF system. (**A**) This panel displays time-averaged streamwise velocity fields across different models. Each row corresponds to a different model output: The top row displays the ground truth obtained from DNS, followed by results from the classical model without Q-Prior, the QIML model using Q-Prior trained on the emulator, and the model using a Q-Prior trained on real superconducting hardware (IQM). (**B**) This panel plots the ensemble energy spectrum 〈E(k)〉 as a function of the spatial wave number *k*. (**C**) This panel displays the time-averaged TKE field for the same four configurations as in (A), using a normalized color scale to enhance visual comparability. (**D**) This panel shows the nondimensional velocity profile *u*^+^ as a function of the wall-normal coordinate *y*^+^ on a logarithmic scale. Results are compared with the empirical log law and DNS reference, using the same validation data as in previous panels.

Figure 6 presents the evaluation of the QIML framework on the TCF dataset. Our ML models perform autoregressive (one frame in, one frame out) inference over a horizon of 1000 time steps. The visualizations span a 2D domain of size 64 × 64 in the *xy* plane. Figure 6A shows a comparison of time-averaged velocity magnitudes *u* across a 64 × 64 domain for the ground truth, the ML model without Q-Prior, and two Q-Prior–enhanced variants: the emulator and real superconducting IQM Quantum Computers (IQM) device. The color bar on the right indicates the magnitude of *u*, with higher values in yellow and lower values in blue. The ground truth (top left) displays smooth, large-scale variations in the velocity field. The model without Q-Prior (top right) introduces noticeable small-scale noise and fails to capture the smooth gradients seen in the true field. In contrast, both Q-Prior (with emulator and with IQM device) models (bottom row) closely match the ground truth, preserving the dominant spatial structures and exhibiting lower spurious variability. Figure 6B plots the ensemble energy spectrum 〈E(k)〉 as a function of the wave number *k* for the raw data (solid blue line), the ML model without Q-Prior (green dashed line), the model with Q-Prior (orange dash-dotted line), and the model with Q-Prior IQM (purple dotted line). The model without Q-Prior deviates from the ground truth at higher wave numbers, with discrepancies arising at smaller spatial scales, which indicates a failure to capture the essential small-scale physical dynamics of the system. As we will demonstrate in the following Discussion, this is a common failure mode for state-of-the-art classical architectures. In contrast, both Q-Prior variants better capture the energy distribution across all scales. Figure 6C compares the normalized TKE fields across a 64 × 64 spatial domain for the ground truth, the model without Q-Prior, and two Q-Prior-based configurations: the emulator and IQM. The ground truth (top left) displays spatially structured regions of elevated TKE, particularly near the domain boundaries, reflecting characteristic flow features. The model without Q-Prior (top right) fails to capture any meaningful TKE patterns, instead producing a near-zero field, which indicates an almost static prediction over time. In contrast, both Q-Prior-–based models (bottom row) recover structured TKE fields that closely resemble the ground truth. These models preserve the dominant energy distribution and capture the spatial organization of turbulent structures more accurately. Figure 6D shows the dimensionless velocity profile *u*^+^ as a function of the dimensionless wall-normal coordinate *y*^+^. Our results are compared with the raw data, log law, and DNS reference. All ML models reproduce the canonical log-law behavior and match well with the reference data, DNS data, and log law.

Overall, the Q-Prior provides a clear improvement in the spatiotemporal prediction of cross sections in TCF, where governing equations for the cross-sectional dynamics are not explicitly defined. This makes it a potentially valuable tool for engineering applications that rely on coupling Reynolds-averaged Navier-Stokes and LES approaches (*62*).

Collectively, these results demonstrate that embedding Q-Priors significantly improves long-term prediction results and statistical alignment of classic ML models in high-dimensional dynamical systems. Even with modest quantum resources, that is, no more than 15 qubits and under 300 parameters (specifically shown in Materials and Methods and Supplementary section S2), our framework captures essential dynamics of high-dimensional flows and enables practical hybrid learning pipelines for scientific applications. By anchoring predictions to Q-Prior, the QIML model improves statistical fidelity and stability, laying the groundwork for practical quantum-classical synergies in the modeling of turbulent and other chaotic physical systems.

## DISCUSSION

### Comparative analysis of QIML against ML baselines

To rigorously quantify the contribution of our QIML framework, we conduct a comparative analysis of our full QIML architecture against key baseline models. The aim is to show that the stability and statistical fidelity observed in our results arise from the Q-Prior guidance rather than being solely attributable to the autoregressive classical ML model’s architecture. Both the QIML framework and the classical baselines are trained on exactly the same raw dataset. The fundamental difference lies in the architectural approach instead of unequal access to data. Here, we consider three primary classes of baselines: (i) a classical state-of-the-art Koopman-based autoregressive ML model trained without the Q-Prior, (ii) neural operators for nonlinear PDEs and chaotic systems such as the FNO and the MNO (*57*), and (iii) a classical-informed ML (CIML) variant in which the same architecture is guided by a classical generative prior (*63*).

**Fig. 7.**
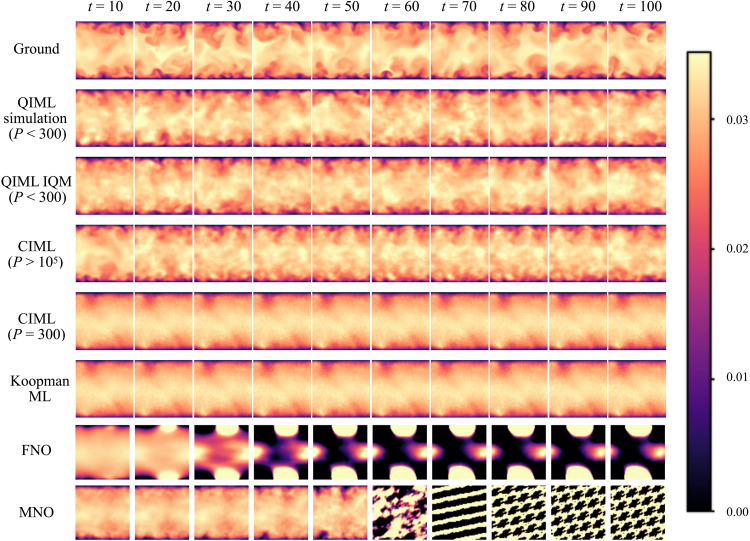
Qualitative comparison of long-term autoregressive predictions for the turbulent channel inflow, with all models evaluated under an identical one-step-in, one-step-out autoregressive rollout. Snapshots of the velocity field are shown at representative predictive time steps *t* from 10 to 100 (from left to right) for the ground truth and for predictions from the QIML (IQM), CIML [variational autoencoder (VAE)], classical Koopman ML, FNO, and MNO models. *P* represents the number of parameters used in Q-Prior or C-Prior. The comparison demonstrates that QIML most consistently preserves the structural features of the ground-truth flow over long prediction horizons. In contrast, most classical baselines either relax toward an overly smooth, time-averaged state or develop nonphysical artifacts during rollout. While the CIML model can reproduce comparable large-scale structures when equipped with a substantially larger parameter budget, it requires more than 400 times as many parameters to approach the performance of QIML. Moreover, after *t* = 20, the CIML predictions display a pronounced locking behavior, in which the flow field exhibits reduced temporal variability and gradually settles into a nearly static configuration. By contrast, QIML shows a more physically consistent temporal evolution, with sustained variability and evolving flow structures over time.

As demonstrated in Fig. 7, our classical model without the Q-Prior consistently fails to maintain long-term stability. While capable of accurate short-term predictions, it inevitably succumbs to error accumulation, leading to prediction divergence and a failure to reproduce the system’s invariant statistical measures. The comparison with neural operators provides a more stringent test. FNOs and MNOs are known for their strong performance in learning resolution-independent solutions to nonlinear PDEs and chaotic systems. We trained both models on the same datasets used for our framework and ensured that both baselines were evaluated under the same autoregressive setting, using a one-step-in, one-step-out rollout in the spatiotemporal domain. Under this regime, however, the FNO and MNO models exhibit the same weakness: Their predictions diverge progressively from the ground truth, failing to preserve the correct long-term spatiotemporal evolution of the system (see Fig. 7). This behavior is particularly notable given their substantial size; both the FNO and MNO use an order of magnitude more trainable parameters than our QIML framework, as detailed in table S2. The underlying issue is well documented (*64*): Standard neural operators are typically trained in a teacher-forcing or multistep-output setting, but when forced into strict one-step autoregressive rollouts, small local errors compound at each iteration. An important observation is that existing FNO and MNO models have demonstrated strong performance in equation-driven PDE systems (*65*), such as 2D Kolmogorov flow, where the governing equations are explicitly known. Nonetheless, in settings such as our cross section of 3D TCF, where no closed-form PDE governs the evolution of the extracted 2D data, these operator-learning models face intrinsic challenges. This highlights that while FNOs and MNOs excel in PDE-constrained environments, their generalization to more complex, data-driven scenarios still needs to be improved.

Furthermore, to rigorously assess the role of the Q-Prior, we conducted a controlled comparison against a high-capacity classical generative baseline. Specifically, a deep variational autoencoder (VAE) (*63*) was trained on the identical TCF dataset to construct a classical prior (C-Prior) with the same output dimensionality as the Q-Prior. When scaled to more than 1.2 × 10^5^ trainable parameters, the VAE-based C-Prior is able to reproduce the coarse-grained energy distribution and large-scale statistics at a level comparable to the Q-Prior (Fig. 7). However, beyond intermediate prediction times, the CIML rollouts exhibit a pronounced tendency toward dynamical saturation, with the predicted flow becoming progressively locked into a quasistationary pattern and displaying reduced temporal variability. These effects accumulate during long-term autoregressive prediction and lead to increased dynamical drift relative to QIML. In addition, systematic deviations emerge in the high-wave number regime, where the C-Prior underestimates the tail of the energy spectrum and fails to faithfully capture fine-scale, high-frequency structures (see section S7). A more direct comparison emerges when the C-Prior is constrained to a parameter budget comparable to that of the Q-Prior (approximately 300 parameters). In this regime, the model collapses to an overly smooth, mean-field representation and fails to recover meaningful small-scale statistics.

This comparative analysis leads to a clear conclusion: The key enabling component of our framework is the Q-Prior integrated into the ML. The long-term instability is a fundamental challenge for even powerful classical architectures such FNOs, MNOs, and even CIML. By providing a physically grounded, memory-efficient statistical regularizer, the Q-Prior confers the necessary stability and long-term fidelity that are otherwise absent. This result underscores the unique and practical advantage of our QIML architecture.

This observation raises a fundamental question: Why does the Q-Prior provide a distinct benefit in modeling chaotic systems? We hypothesize that the answer lies in the intrinsic structure of chaotic data, which often exhibits complex, nonlocal statistical dependencies—structures that may be functionally analogous to quantum entanglement or contextuality. These global correlations, inherent to the invariant measure of a chaotic attractor, are notoriously difficult for classical generative models to capture efficiently due to their reliance on factorized or locally conditioned distributions (*32*). In contrast, quantum circuits naturally encode such correlations through entangled amplitudes, enabling the Q-Prior to compactly represent statistical features that span multiple scales and df. We posit that quantum generative models may thus offer a provable advantage in learning the invariant measure of systems with these characteristics. A theoretical motivation and more discussions are presented in sections S8 and S10.

### Q-Prior memory efficiency

A defining feature of the proposed framework is its capacity to extract high-dimensional physical information using significantly fewer trainable parameters than conventional deep learning models. The Q-Prior, operating on only 10 to 15 qubits and comprising fewer than 300 trainable parameters, successfully captures invariant distributions over spatial domains with dimensionality exceeding 104. In contrast, a classical neural network capable of learning an equally expressive distribution over the same domain would require at least $O\left(10^{4}\right)$ parameters, assuming a minimal fully connected architecture with comparable granularity. For example, on a grid of *D* = 2^10^ = 1024 spatial points, the lightest fully connected network that can approximate an arbitrary probability map with standard universal-approximation accuracy must allocate $O\left(D^{2}\right)$ weights (*66*, *67*). Even with an aggressively thinned hidden layer, this still amounts to ~2.5 × 10^5^ tunable parameters—three orders of magnitude more than the 300 parameters used by our 10-qubit Q-Prior (see section S2). Moreover, classical models lack access to quantum entanglement, limiting their ability to compactly encode nonlocal correlations and higher-order statistical dependencies prevalent in turbulent or chaotic systems.

However, having a parameter-efficient quantum representation is only the first step; leveraging it to guide a classical ML model is a distinct and significant challenge. We address this by designing a composite loss function that seamlessly integrates the statistical information from the Q-Prior into the classical model’s training process. This enables a true “quantum-informed” learning paradigm where the quantum component actively regularizes the classical dynamics, leading to superior results as demonstrated in this work. As shown in the Table 1, the successful implementation of this hybrid integration unlocks a further, highly practical potential of quantum computing: memory efficiency in data storage and transfer.

**Table 1.** Quantum resources and storage compression achieved by the Q-Prior.

Dynamical system	Raw data	Q-Prior	Compression
	(Full set)	(Full set)	(Ratio)
Kuramoto-Sivashinsky	300 megabytes	0.25 megabytes	≈1.2 × 10^3^:1
2D Kolmogorov flow	400 megabytes	0.40 megabytes	≈10^3^:1
Turbulent channel flow	500 megabytes	2.3 megabytes	≈2.0 × 10^2^:1

Specifically, for the turbulent-channel dataset, retaining all midplane snapshots in double precision requires roughly 5.0 × 10^2^ megabytes. Archiving the corresponding Q-Prior checkpoint for each snapshot (4.3 kilobytes per file) occupies only 595 × 4.3 kilobytes ≈ 2.5 megabytes, a compression greater than two orders of magnitude while preserving the relevant flow statistics. Comparable savings are obtained for Kolmogorov flow (~180 parameters, ~1.4 kilobytes per file, ~0.44 megabytes in total) and KS data (~120 parameters, ~1 kilobyte per file, ~ 1.4 megabytes in total). Full storage calculations are shown and reported in section S8. This marked savings make the Q-Prior attractive whenever data are expensive to generate or archive while still providing a statistically faithful, low-parameter regularizer for the classical Koopman ML.

Crucially, this provides a blueprint for future hybrid computing infrastructures where the quantum processors might be colocated with every classical computer. Instead of transferring voluminous raw datasets, which can amount to hundreds of gigabytes or more, one would only need to transfer the compact Q-Prior. As our results show, this corresponds to a data storage reduction of over two orders of magnitude. Furthermore, this vision is made practical by a key finding that directly addresses the number of measurement shots, a widely scrutinized issue for NISQ-era algorithms. While the results presented in this work are based on a high shot count (20,000 per iteration) to demonstrate the framework’s maximum potential, we found that the classical model does not require a perfectly resolved, exponentially large distribution to be effective. Reducing the measurement count to 8000 shots had a negligible impact (less than 5%) on the final model’s performance, showing that an approximate prior is sufficient to act as a powerful guiding signal. Crucially, the Q-Prior is trained only once in an offline stage rather than being reevaluated during every rollout or trained iteratively within the ML framework such as QEML. This one-time training greatly reduces the overall computational burden and differentiates our approach from iterative hybrid quantum-classical schemes that require repeated quantum calls throughout the learning process. Ideally, the sample complexity for estimating gradients of Born machines scales polynomially with the number of qubits (*68*), ensuring that the shot overhead remains manageable even as we scale to larger systems. Thus, QIML establishes a viable and economically attractive framework: A central, high-fidelity quantum processor performs the intensive, one-time training of a master Q-Prior, whose compact parameters are then distributed at low cost to remote systems. This paradigm may serve as a template for future cloud quantum platforms, where the quantum processors provide powerful statistical guidance and data support for large-scale classical models.

### On the nature of practical quantum advantage in QIML

The term “quantum advantage” must be approached with caution, particularly in the context of quantum computing and quantum ML (*68*, *69*). As recent analyses have highlighted, many claims of quantum advantage are later matched or surpassed by sophisticated classical algorithms, revealing that the initial gap was due to the immaturity of classical methods rather than a fundamental quantum superiority (*68*). With the exception of algorithms such as Shor’s (*70*), which leverage the fundamental nature of quantum mechanics in a provably superior way, many claims of advantage are eventually challenged. Even powerful algorithms such as the HHL algorithm have faced scrutiny (*46*), with new classical works emerging that achieve comparable performance in specific contexts (*68*). We do not assert that QIML holds an unassailable advantage. Instead, we posit that its effectiveness stems from a foundational quantum principle that, in this application, has not been classically replicated. There is growing evidence that certain probability distributions are exponentially hard to represent or learn classically if they embody forms of contextuality or nonlocal correlations analogous to those in quantum states (*32*).

The foundational advantage exploited by our Q-Prior stems from the ability of an *n*-qubit quantum state to represent a classical vector in a 2*^n^* dimensional space, which is considered a genuine quantum advantage (*50*). This provides an exponential advantage in information storage capacity. This type of advantage has found recent applications in quantum ML, such as for computing quadratic functions of data (*71*). However, this potential is famously constrained by Holevo’s bound, which dictates that only *O(n)* classical bits of information can be reliably extracted from an *n*-qubit state through measurement (*48*). If a full reconstruction of the classical information were required, then the need for an exponential number of measurements would negate the storage advantage entirely.

In ergodic theory, the invariant measure governs the long-time statistical behavior of chaotic dynamics, although individual trajectories diverge exponentially (*72*). The central insight of the QIML framework is to circumvent Holevo’s bound by extracting only this essential invariant measure without reconstructing the full dataset, yielding a compressed yet sufficient representation of the system. This low-dimensional representation is precisely what is needed to regularize the classical ML, guiding it away from nonphysical trajectories without needing to know every detail of the system’s state. Therefore, the advantage demonstrated here is a representational and memory advantage that translates directly into a performance advantage. The theoretical basis for this advantage is detailed in section S8.

Memory efficiency: The Q-Prior leverages quantum entanglement to capture complex, nonlocal correlations within the invariant measure using a few parameters. This translates to a marked, orders-of-magnitude reduction in data storage, as quantified in Table 1.

Performance gain: This compact and physically rich representation provides the crucial regularization that leads to long-horizon stability and statistical fidelity, a task at which larger, more complex classical models such as FNO and MNO demonstrably fail for this class of problems.

While it is an open question whether other classical models could be adapted to leverage the Q-Prior as effectively, our results suggest that the codesign of the QIML architecture is key. We anticipate that our results may inspire new classical approaches. For instance, using large generative models to learn the invariant measure could achieve comparable predictive performance. However, we argue that such methods are unlikely to replicate the foundational source of the advantage demonstrated here: the physical ability to logarithmically compress high-dimensional data and extract the necessary guiding information without an exponential measurement cost. This is possible because the invariant measure appears to be a sufficiently rich representation of the essential physics of chaos. Capturing its key features provides the necessary guidance to unlock the observed gains in performance and, crucially, in memory efficiency. This efficiency is a direct, physical consequence of the quantum representation, and while our theoretical understanding of the measure’s precise role is still developing, the empirical results provide compelling evidence for this paradigm’s value.

Another notable point is that much of that research begins with a search for theoretical advantage, often struggling to connect with practical, “useful” applications (*68*). In contrast, our investigation was born from a practical necessity: to solve the long-horizon prediction problem for chaotic systems, where leading classical methods fail. By showing that a 10-qubit quantum device can provide a unique resource, this work provides a compelling example of a practical quantum advantage and motivates the further development of both quantum and classical algorithms for scientific ML.

### Limitations, immediate, and future impact of QIML

To reduce the impact of hardware noise and enable stable training, the quantum generator is optimized separately and remains fixed during the training of the classical model. This architectural constraint arises from the limited scalability and noise of NISQ superconducting devices, which make full end-to-end quantum training infeasible at present. For near-term work, further improvements are possible within this offline training paradigm. While current Q-Priors are limited by qubit count, circuit depth, and hardware noise, deeper architectures with structured entanglement (e.g., hardware-efficient ansätze) may further improve expressivity for highly anisotropic distributions. Besides, while this work demonstrates improvements in learning from digitally generated high-dimensional dynamical system data, we acknowledge the inherent limitations of the digital computing part of our method in fully capturing the true continuum behavior of high-dimensional dynamical systems (*4*–*7*). Our ground truth here is, by definition, a 64-bit floating-point approximation. Future work will explore the use of analog computation to provide a more direct comparison with the true invariant measures, thus offering a more rigorous validation of our approach against the ultimate ground truth.

Although there are limitations from NISQ hardware, in contrast to prevailing skepticism about the immediate applicability of quantum learning algorithms (*31*, *69*), QIML demonstrates that even current quantum devices can contribute meaningfully to the modeling of high-dimensional dynamical systems. Without resorting to fault-tolerant quantum computing (FTQC) hardware, lightweight NISQ-based quantum modules enhance classical ML models by providing data-driven Q-Priors that efficiently capture complex statistical structures and nonlocal correlations—features that are otherwise challenging for classical methods to acquire with comparable memory efficiency. The resulting gains in statistical fidelity and long-horizon stability, coupled with a substantial reduction in data storage requirements, point to an immediate and practical role for near-term quantum resources in scientific ML. The compact 10- to 50-qubit Q-Prior presented here exemplifies how such modules can be effectively integrated today.

**Fig. 8.**
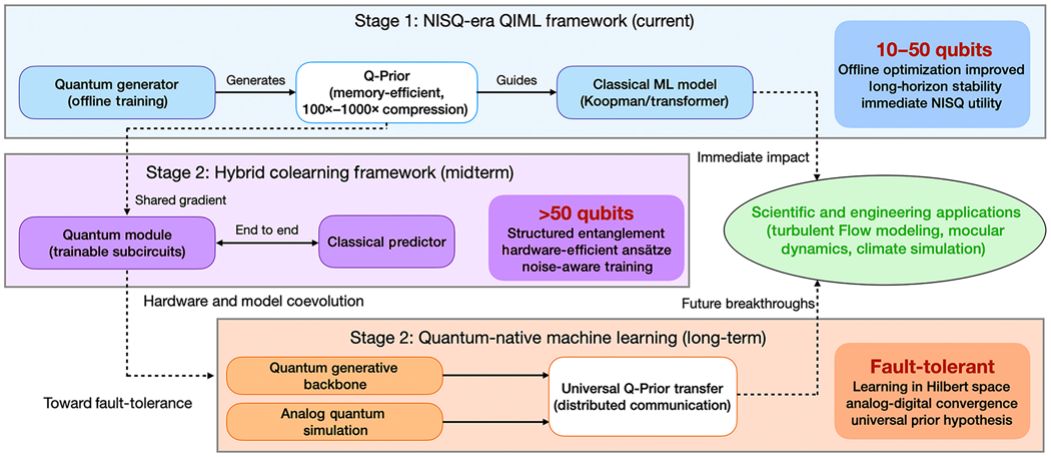
Conceptual roadmap of the QIML paradigm. Stage 1 shows the current NISQ-era QIML framework in which an offline-trained quantum generator produces a memory-efficient Q-Prior that guides the classical model. Stage 2 presents a hybrid colearning QIML framework with shared gradients, structured entanglement, and noise-aware training. Stage 3 outlines quantum-native ML with a quantum generative backbone and analog quantum simulation that enable universal Q-Prior transfer for distributed communication. The diagram highlights the immediate impact on scientific and engineering applications and future breakthroughs through compact, kilobytes-scale Q-Prior transfer between supercomputers and quantum computers.

Looking forward, the QIML architecture establishes a strategic roadmap bridging the NISQ era and the future of FTQC devices. In the near term, we identify a practical operational envelope for QIML involving approximately 50 qubits with circuit depths kept below 20 layers; this configuration balances the representational capacity required for complex flows against the coherence limits of current hardware. This defines our immediate trajectory for scaling up to high-dimensional geophysical datasets, specifically the European Center for Medium-Range Weather Forecasts data, as delineated in stage 2 of Fig. 8. Beyond this, we envision a unified computational paradigm that integrates digital QIML with analog quantum verification mechanisms to tackle industrial-scale turbulence. As illustrated in stage 3 of Fig. 8, this hybrid ecosystem, combining quantum generative priors with classical high-performance computing, serves as a blueprint for realizing practical quantum advantage in computational fluid dynamics and climate science.

Furthermore, this work opens up more exciting avenues for future research. A key question is how the framework performs with sparse or partial datasets, a common scenario in real-world applications where obtaining a complete ground-truth solution is impossible. Our current results are already promising in this regard, as the Q-Priors were successfully trained using only a subset of the available data (see section 2) while still providing effective guidance. This suggests the potential for a more profound, out-of-distribution generalization (*73*). For instance, future work could explore whether a single Q-Prior, trained on the invariant measure of one system, could successfully regularize a model for a different system that shares similar statistical properties but has different underlying dynamics. The successful demonstration of such a “universal” prior would have significant implications, potentially allowing one Q-Prior to guide multiple ML models, further enhancing the practical value and efficiency of the QIML paradigm.

As quantum hardware continues to advance, in terms of qubit count, coherence time, and error mitigation, the prospect of replacing classical generative backbones with fully quantum circuits becomes increasingly viable. In addition, this hybrid architecture lays the groundwork for a future paradigm of highly efficient information transfer within large-scale infrastructures. As quantum devices advance, one can envision a scenario where highly compressed Q-Priors, containing essential physical statistics, are transferred between compute nodes in place of voluminous classical datasets, which are riddled with spurious correlations (*74*). This mechanism offers a scalable solution for future large-data training and provides a foundation for progressively more capable quantum-native ML models as the hardware matures. Thus, the parameter and memory efficiency demonstrated in this work suggest a pathway for Q-Priors to ultimately influence entire sections of large classical models.

## MATERIALS AND METHODS

### Quantum-informed ML

In this section, we present the QIML architecture that leverages a quantum-classical hybrid approach for ML-based modeling of high-dimensional dynamical systems. Our QIML scheme is a specialized form of hybrid quantum-classical ML, which is illustrated in Fig. 1 and fig. S1. Rather than combining quantum and classical modules through layered architectures or repeated variational optimization, QIML uses a quantum generative model as a statistical prior, exploiting the structure of Hilbert space to constrain and regularize a classical predictor. The workflow commences with the data generation stage (Fig. 1A), where we use a numerical solver on a classical computer to simulate complex high-dimensional flows, producing the necessary training, validation, and testing datasets. Subsequently, in the quantum ML phase (Fig. 1B), a quantum generator is implemented on a quantum computer to learn the underlying physical invariant distribution, referred to as the Q-Prior. Last, this Q-Prior is used to inform and regularize the classical ML model during training (Fig. 1C), guiding it toward long-term statistical consistency with the target dynamical system. We begin by introducing the ML component, which is based on a Koopman operator formulation. The Koopman operator offers a linear representation of nonlinear dynamical systems by acting on observables instead of directly on the state variables. In this framework, the evolution of the system is captured not in the original state space but in a lifted function space where the dynamics become linear. Consider a discrete-time dynamical system

$$x_{t+1}=f\left(x_{t}\right)$$ (4)

where $x_{t}\in {\mathbb{R}}^{n}$ with *n* denotes the dimension of the state space at time *t* and *f* is a generally nonlinear transition function. To enable autoregressive prediction and long-term rollouts, we aim to find a mapping from the high-dimensional space to a latent space where a linear operator governs the time evolution. The measure-preserving property in the latent space is particularly important for capturing the statistical properties and invariant measure μ of high-dimensional dynamical systems. The Koopman operator **K** acts on observables $g:{\mathbb{R}}^{n}\to \mathbb{C}$ such that

(Kg)(x)=g[f(x)] (5)

While **K** is linear, our implementations seek finite-dimensional approximations by learning a set of observables whose evolution under **K** is closed and linear. Next, we approximate the Koopman operator using a neural network; we define an encoder $\phi :{\mathbb{R}}^{n}\to {\mathbb{R}}^{l}$, with *l* denotes the latent dimension, which maps the system state into a latent (feature) space, and a decoder $\psi :{\mathbb{R}}^{l}\to {\mathbb{R}}^{n}$ that reconstructs the original state. Unlike generic autoencoders primarily focused on data reconstruction, our Koopman operator neural network is specifically designed to learn a linear, norm-preserving evolution in the latent space, which is crucial for maintaining the long-term stability and physical fidelity of high-dimensional dynamical systems. Our detailed autoencoder-decoder architecture description can be found in section S7 for 1D and 2D cases. The dynamics in the latent space are modeled by a linear operator $K\in {\mathbb{R}}^{l\times l}$, such that

$$z_{t}=\phi \left(x_{t}\right),z_{t+1}={\mathrm{Kz}}_{t},{\hat{x}}_{t}=\psi \left(z_{t}\right)$$ (6)

where **z***_t_* is the encoded latent state and ${\hat{x}}_{t}$ is the reconstructed state. To preserve the long-term statistical behavior of the system and approximate the spectral properties of a measure-preserving dynamical system, we constrain the operator **K** to be unitary, i.e., $K^{⊤}K=I$ (*54*, *75*, *76*). This ensures that the Koopman dynamics in the latent space are norm preserving and stable under long-term rollouts. In this work, unitarity is imposed by adding a regularization term to the loss function of the form $L_{\text{unitary}}=∥K^{⊤}K-I{∥}^{2}$. The Koopman operator **K** evolves the system in a latent Hilbert space of encoded observables, enabling a linear and spectrally coherent representation of the dynamics. However, the preservation of long-term statistical properties in this latent space does not automatically guarantee consistency with the statistical structure of the original physical space.

To address this, we integrate a sample-based quantum generator, which approximates the invariant distribution Q-Prior $p_{\theta }\left(x\right)$ of the system. This quantum-learned prior is used as a statistical regularizer and physically informed guidance during the classical ML training, guiding the model to produce predictions that are not only temporally coherent but also statistically consistent with the system’s long-term behavior (see the following sections). This integration is achieved by incorporating the Q-Prior into a composite loss function that combines reconstruction fidelity with statistical alignment metrics, which is shown below.

While the Koopman operator is introduced using a generic state variable, in this work, we focus on predicting physical states **u**. We use the Koopman operator to predict the next time frame of the physical states ${\hat{u}}_{t+1}$. The training of our model is guided by a composite loss function. We define its components below.

First, to ensure predictive accuracy, we use a standard reconstruction loss $L_{\text{recon}}$, which measures the MSE between the predicted state ${\hat{u}}_{t+1}$ and the ground truth $u_{t+1}$

$$L_{\text{recon}}={\left\|{\hat{u}}_{t+1}-u_{t+1}\right\|}^{2}$$ (7)

Second, we include the unitary regularization term $L_{\text{unitary}}$ as previously discussed

$$L_{\text{unitary}}={\left\|K^{⊤}K-I\right\|}_{F}^{2}$$ (8)

where **I** is the identity matrix and $∥\cdot {∥}_{F}$ denotes the Frobenius norm. Last, we incorporate the Q-Prior, $p_{\theta }\left(x\right)$, as a statistical constraint. This is achieved through two complementary metrics. The Kullback-Leibler (KL) divergence $L_{\text{KL}}$ captures low-order, information-theoretic discrepancies between the empirical distribution of predicted states $\hat{q}\left(x\right)$ and the Q-Prior

$$L_{\text{KL}}=D_{\text{KL}}\left[\hat{q}\left(x\right)∥p_{\theta }\left(x\right)\right]$$ (9)

To align higher-order statistical moments, we also include an MMD term $L_{\text{MMD}}$

$$L_{\text{MMD}}={\left\|E_{x∼\hat{q}\left(x\right)}\left[\phi \left(x\right)\right]-E_{x∼p_{\theta }\left(x\right)}\left[\phi \left(x\right)\right]\right\|}_{H}^{2}$$ (10)

These individual components are combined into a single training objective, where the Q-Prior terms are weighted by empirically selected hyperparameters $\lambda_{\text{KL}}$ and $\lambda_{\text{MMD}}$

$$L_{\text{total}}=L_{\text{recon}}+L_{\text{unitary}}+\lambda_{\text{KL}}L_{\text{KL}}+\lambda_{\text{MMD}}L_{\text{MMD}}$$ (11)

The full QIML framework is demonstrated in Algorithm 1. All models are implemented in PyTorch with fully differentiable integration of Q-Priors. Architectural details for predicting 1D and 2D systems are provided in section S7.

**Algorithm 1.**
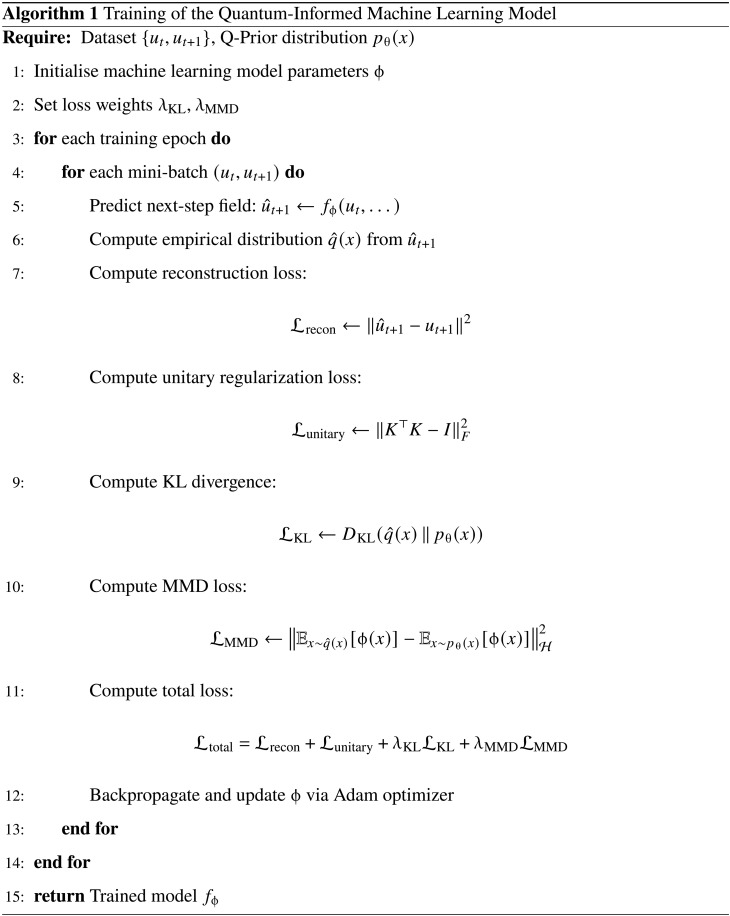
Training of the QIML model.

### Invariant measure for high-dimensional dynamical systems

The concept of an invariant measure originates from the field of ergodic theory and has become a fundamental tool in the study of dynamical systems. First formalized in the context of measure-preserving transformations by Birkhoff and von Neumann (*77*, *78*) in the early 20th century, invariant measures capture the long-term statistical behavior of systems evolving under deterministic rules.

An invariant measure describes how a dynamical system spends its time in different regions of its state space over the long term. For a system evolving under a map *T*, the invariant measure μ(*x*) satisfies

$$\mu \left[T^{-1}\left(A\right)\right]=\mu \left(A\right)$$ (12)

which means that the total probability of the system being in a region *A* remains the same after one step of evolution. This property reflects a kind of statistical balance: Although individual trajectories may change, the overall distribution stays stable. In our work, we aim to capture this long-term behavior by ensuring that the model’s predictions remain consistent with a reference distribution, which we learn using a quantum generator.

In turbulent flows, these invariant measures typically manifest as conserved distributions, such as velocity probability, TKE, or energy spectra that encode physical long-term steady states. Capturing these invariant features is essential for ensuring robustness in extrapolative prediction regimes, where a classical ML method often suffers from gradient explosion, mode collapse, or long-term error accumulation (*79*).

### The quantum generator to learn the Q-Prior

**Fig. 9.**
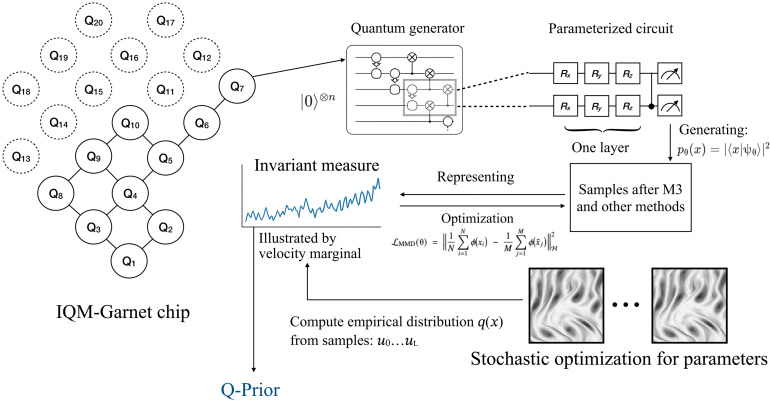
Schematic illustration of the Q-Prior generation within the QIML framework. The quantum circuit functions as a Born machine, initialized in a fixed vacuum state ${|0\rangle }^{⊗n}$. No classical data are encoded into the input state amplitudes. The circuit U(θ) evolves the state to generate a wave function $|\psi_{\theta }\rangle$, and the measurement statistics in the computational basis, $p_{\theta }\left(x\right)={|\langle x|\psi_{\theta }\rangle |}^{2}$, form the model distribution. The classical turbulent data samples serve solely as the target for calculating the loss, driving the optimization of parameters θ to θ^*^.

We develop a quantum-informed learning component in which a quantum generator is used to directly learn invariant statistical properties from high-dimensional physical observations. The schematic illustration of the Q-Prior generation is shown in Fig. 9. The generator defines a parameterized quantum state $|\psi_{\theta }\rangle$ on an *n*-qubit register, which serves as a generative model over a discretized spatial domain. It does not encode a specific state (e.g., a velocity field) into the circuit. Instead, by observing many snapshots of the system, it learns to reproduce its underlying invariant probability distribution. Specifically, each computational basis state $x\in {\left\{0,1\right\}}^{n}$ here is mapped bijectively to a spatial coordinate in the simulation grid. The corresponding amplitude $\langle x|\psi_{\theta }\rangle$ is optimized so that its squared magnitude approximates the normalized velocity magnitude at that point. Although the circuit does not encode classical inputs directly via amplitude encoding, it constructs a quantum state whose Born distribution approximates the invariant measure Q-Prior of the system. The resulting Born distribution is given by

$$p_{\theta }\left(x\right)={|{\langle x|U\left(\theta \right)|0\rangle }^{⊗n}|}^{2}$$ (13)

where *U*(θ) is a parameterized unitary circuit composed of *L* layers, each consisting of single-qubit rotations and entangling operations. Each computational basis state represents a discrete spatial point, and the quantum amplitudes are trained to reflect the empirical distribution of local velocity magnitudes. This provides a compact, data-driven mapping from physical observables into the Hilbert space. The exponential size of this space, combined with the use of entangling gates, provides a natural mechanism to encode the complex, nonlocal correlations inherent in chaotic systems. This feature provides a quantum advantage rooted in the ability of quantum systems to represent high-dimensional classical data with exponential efficiency (*68*). This enables it to learn the required statistical properties using exponentially fewer parameters than would be required by a classical model (*80*). Furthermore, by leveraging the vast Hilbert space and entanglement, the quantum circuit can capture the complex, nonlocal correlations inherent in such systems efficiently (*50*). Further details can be found in Discussion and section S1.

As shown in Algorithm 2, the quantum circuit is trained as a quantum generative model. Each computational basis state $x\in {\left\{0,1\right\}}^{n}$ is mapped uniquely to a spatial location **r***_x_* in the discretized velocity field, and its target value is the corresponding velocity magnitude *u*(**r***_x_*). Because the underlying probability distribution over spatial locations is unknown, we adopt a sample-based training strategy that minimizes the MMD loss

$$L_{\text{MMD}}\left(\theta \right)={\left\|\frac{1}{N}\sum_{i=1}^{N}\phi \left(x_{i}\right)-\frac{1}{M}\sum_{j=1}^{M}\phi \left({\tilde{x}}_{j}\right)\right\|}_{H}^{2}$$ (14)

Here, ${\left\{x_{i}\right\}}_{i=1}^{N}$ denotes the batch of empirical samples drawn from the reference velocity field, while ${\left\{{\tilde{x}}_{j}\right\}}_{j=1}^{M}∼p_{\theta }\left(x\right)$ are samples generated by the quantum circuits. Because the MMD objective is expressed solely through kernel evaluations on finite sample sets, optimization of the generator requires no closed-form expression for the target probability density. This sample-based objective is particularly advantageous for high-dimensional chaotic flows, where the invariant measure is available only as a time-ordered set of simulation snapshots rather than an explicit probability density. By aligning the quantum generator’s samples with these numerical trajectories, the model infers the underlying measure without ever needing a closed-form expression.

The trained quantum distribution $p_{\theta }\left(x\right)$ thus can serve as a data-driven prior capturing the invariant measure of the underlying physical process. Unlike classical regularizers based on fixed heuristics, the quantum generator constructs this Q-Prior through optimization on empirical data.

All quantum circuits are implemented using hardware-efficient structures, which are detailed in section S2. Training is carried out on noiseless simulators and on superconducting quantum hardware, with circuit transpilation, error mitigation, and device-specific calibration performed using the Qiskit runtime. Further details and the optimization methods are provided in sections S1 and S2.

**Algorithm 2.**
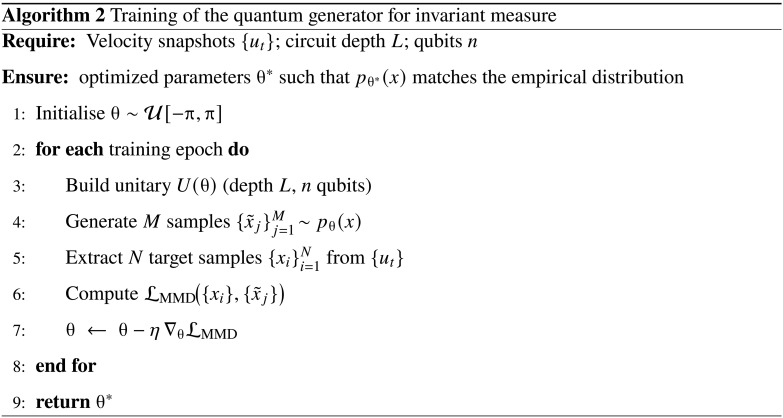
Training of the quantum generator for invariant measure.

### Quantum hardware implementation

The sample-based quantum generator model was trained using both emulators on classical computers and real superconducting quantum processors. A classical emulator here is a program running on a conventional computer that simulates the mathematical operations of an ideal, noise-free quantum device. In this work, these emulations were supported by the PennyLane and Qiskit software packages. The practical constraints of current NISQ-era hardware, which require cryogenic operating temperatures and are subject to frequent maintenance and recalibration, make continuous, large-scale training across numerous datasets challenging. For this reason, we adopted a strategic approach here. The first two systems (KS and Kolmogorov flow) were studied on the classical emulator to establish the Q-Prior’s efficacy in an ideal, noise-free setting. We then reserved the use of the real quantum hardware for the most complex and challenging dataset, which is the TCF, precisely because this is the case where leading classical methods fail, providing the most stringent test for the QIML framework’s practical utility and robustness under realistic noise conditions. To ensure effective execution on near-term quantum hardware and to manage the impact of noise on the quality of Q-Priors, this study constrains the total number of trainable parameters and adopts conservative circuit configurations. Specifically, the number of qubits is 10 to 15 on the emulator and 10 on the hardware, with circuit depths ranging from three to eight layers, resulting in fewer than 300 trainable parameters in total (see table S3). Hardware experiments were mainly conducted on IQM’s 20-qubit superconducting chip (Garnet) (*81*), using qubits 1 to 10 as the active subset, supported by Qiskit and IQM-Qiskit. Further discussion on hardware implementation and sensitivity is provided in section S2. To ensure scalability, the quantum emulation and hardware backend were integrated into a GPU-accelerated classical training loop on NVIDIA A100 nodes provided by the BEAST GPU cluster (Leibniz Supercomputing Centre). Gradient evaluations, loss computations, and optimizer updates were performed classically and synchronized with the quantum hardware at each iteration. This setup constitutes an applied demonstration of embedding quantum devices into scientific ML pipelines. The used quantum circuit architecture consisted of alternating layers of parameterized *R_x_* rotations and hardware-native entangling gates. The same circuit structure was used across all experiments without dataset-specific tailoring, ensuring general applicability. Although performance could potentially be improved through customized ansatz design, our results demonstrate that the proposed quantum-informed framework is compatible with any hardware-efficient quantum circuit. This adaptability enables the integration of diverse circuit architectures without modifying the overall training pipeline, suggesting broad utility across problem domains and quantum backends.

The Q-Prior is generated via a one-time, offline training process. This training consists of a total of 50 epochs. Following the completion of this training, the quantum circuit is not used during the main classical model’s training loop. For each of the 50 training epochs, the circuit was sampled with 20,000 shots to ensure a high-fidelity statistical distribution. Readout errors were mitigated using IQM’s native transpiler and the M3 measurement mitigation toolkit (*82*). While we used this number of shots to ensure maximum stability and demonstrate the framework’s potential, this is not a strict requirement for its effectiveness. In separate tests, we reduced the measurement count to 8000 shots and found that the impact on the final model’s performance was less than 5%, indicating that the Q-Prior remains effective. Nevertheless, to present the best possible outcomes in this work, all results and figures are based on data obtained from 20,000 shots per iteration. Optimization was performed using L-BFGS (*83*), Adam (*84*), and COBYLA (*85*), selected on the basis of convergence stability and noise robustness. To prevent mode collapse, such as excessive sampling concentration on the ∣0〉 state, runtime monitoring and redundancy checks were incorporated into the training loop. Despite operating with only 10 to 15 qubits, less than 20 circuit depth, and 300 quantum gates, the Q-Prior successfully captured the structure of invariant distributions over a 1000D to 60,000D Hilbert space, demonstrating the effectiveness of Q-Priors in modeling high-dimensional systems. To contextualize the practical feasibility of our approach, table S1 compares the resource budget of the QIML framework against established quantum algorithms such as VQE (*33*) and HHL, highlighting its significantly lower requirements for circuit depth and measurement overhead. Further discussions of the error mitigations, experiment results, and ablations are provided in sections S2 and S7.

## Supplementary Materials

### The PDF file includes:

Supplementary Text Sections S1 to S10 Figs. S1 to S9 Tables S1 to S5 References
