# Supplementary material for: Quantum-informed machine learning for predicting spatiotemporal chaos with practical quantum advantage
Source: Sci Adv. 2026 Apr 17;12(16):eaec5049. doi: 10.1126/sciadv.aec5049 (PMC13089341; doi:10.1126/sciadv.aec5049)
Supplement: Supplementary file 1 — Supplementary Text Sections S1 to S10 Figs. S1 to S9 Tables S1 to S5 References [file sciadv.aec5049_sm.pdf]

Science Advances
AAAS

Supplementary Materials for

Quantum-informed machine learning for predicting spatiotemporal chaos with practical quantum advantage

Maida Wang *et al.*

Corresponding author: Peter V. Coveney, p.v.coveney@ucl.ac.uk

Sci. Adv. **12**, eaec5049 (2026)
DOI: 10.1126/sciadv.aec5049

This PDF file includes:

Supplementary Text
Sections S1 to S10
Figs. S1 to S9
Tables S1 to S5
References

S1 Quantum Computing and Quantum Generator

S1.1 Quantum Computing

Quantum computing operates on the principles of quantum mechanics, allowing for a novel class of information processing paradigms. The fundamental unit of quantum information is the *qubit*, which exists in a superposition of classical states. Formally, a single qubit can be represented as

$$|\psi\rangle = \alpha|0\rangle + \beta|1\rangle, \tag{S1}$$

where $\alpha, \beta \in \mathbb{C}$ and $|\alpha|^2 + |\beta|^2 = 1$. Upon measurement in the computational basis, the qubit collapses to state $|0\rangle$ or $|1\rangle$ with probabilities $|\alpha|^2$ and $|\beta|^2$, respectively. In an n-qubit system, the global state resides in a 2^n-dimensional Hilbert space, allowing the compact representation of complex, high-dimensional distributions.

S1.2 Quantum Machine Learning

Recent progress in quantum computing has spurred the rapid emergence of QML as a promising interdisciplinary field at the intersection of quantum information science and statistical learning. QML algorithms are designed to exploit quantum phenomena, such as entanglement, interference, and superposition, to augment learning efficiency or representational capacity in ways that may surpass traditional counterparts. A variety of model classes have been developed within this paradigm, including quantum autoencoders (*86–88*), which perform lossy compression and denoising on quantum data using a reduced number of qubits, and quantum Boltzmann machines (*89, 90*), which offer a framework for generative modelling and state tomography through quantum sampling of thermal distributions.

Another line of work explores quantum generative adversarial networks (QGANs) (*91–93*), which have been employed to emulate quantum systems and approximate entangled states through adversarial training mechanisms. In the context of supervised learning, quantum kernel methods (*94–96*) have been proposed to enhance the expressive power of traditional classifiers by mapping data into high-dimensional Hilbert spaces via quantum feature maps.

Despite the theoretical potential of QML models, their application to real-world, non-quantum datasets remains in its infancy. Many benchmark results have been demonstrated, primarily in

controlled settings with synthetic or quantum-native inputs. Notably, Huang et al. (*50*) recently showed that a 40-qubit quantum processor could infer global properties of a data distribution using exponentially fewer samples than traditional learners, albeit in a tightly constrained regime. While such results mark a significant step forward, the challenge of applying QML to traditional scientific tasks with high-dimensional structure and dynamical complexity remains open.

Such matters motivate us to develop a quantum-informed machine learning framework (QIML) that incorporates quantum components as functional modules within traditional workflows. In particular, demonstrating the ability of QML models to learn nontrivial statistical priors or invariant structures from real data, such as those arising in fluid dynamics or nonlinear PDEs, would provide a critical proof-of-concept for their practical relevance. To this end, and to circumvent the significant challenge of encoding high-dimensional classical data into quantum states, our QIML framework utilizes a sample-based quantum circuit as its generative module.

S1.3 Sample-based Quantum Generator

Quantum generative models have shown great potential recently (*97,98*), where quantum circuits are trained to learn quantum states or model traditional probability distributions. The quantum generator employed in this work is a sample-based model, with an architecture based on the quantum circuit Born machine. Given a parameterized quantum circuit $U(\boldsymbol{\theta})$, initialized from the all-zero state $|0\rangle^{\otimes n}$, the probability of observing a bitstring $x \in \{0,1\}^n$ upon measurement is governed by the Born rule:

$$p_{\boldsymbol{\theta}}(x) = |\langle x|U(\boldsymbol{\theta})|0\rangle^{\otimes n}|^2. \tag{S2}$$

These probabilities define an implicit generative model from which samples can be drawn directly via quantum measurement, without requiring explicit likelihoods or tractable gradients.

Quantum generators have demonstrated utility in modelling structured datasets in domains such as quantum chemistry, generative learning, and combinatorial optimisation. However, their application to traditional physical systems, particularly those governed by nonlinear PDEs, remains limited. This is despite the deep structural parallels between quantum mechanics and classical dynamical systems: both evolve within high-dimensional Hilbert spaces, exhibit conservation laws, and are constrained by symmetry principles. These analogies motivate the integration of quantum-

generated priors into classical scientific machine learning pipelines.

The sample-based quantum generator implementation in this work is distinguished by its likelihood-free training approach: a parameterized quantum circuit encodes a probability distribution through measurement statistics, without ever writing down an explicit probability mass function. We describe the full circuit ansatz and its Born-rule output distribution in Methods C; the Supplementary material therefore omits the detailed equations and simply points readers to that section for implementation specifics.

Empirical and model sample sets. A batch of empirical samples $\{x_i\}_{i=1}^{N}$ is obtained by converting the normalized velocity magnitudes $v(\mathbf{r})$ into a categorical distribution over spatial indices and drawing N indices. Model samples $\{\tilde{x}_j\}_{j=1}^{M} \sim p_{\theta}$ are produced by executing the circuit M times on the quantum processor. These two finite sample sets constitute the sole inputs to the training loss.

MMD training objective. Using a characteristic kernel $k(x, x') = \langle \phi(x), \phi(x') \rangle$ the maximum-mean-discrepancy (MMD) between the empirical and model samples is

$$\mathcal{L}_{\mathrm{MMD}}(\theta) = \frac{1}{N(N-1)} \sum_{i \neq i'} k(x_i, x_{i'}) + \frac{1}{M(M-1)} \sum_{j \neq j'} k(\tilde{x}_j, \tilde{x}_{j'}) - \frac{2}{NM} \sum_{i,j} k(x_i, \tilde{x}_j), \qquad \text{(S3)}$$

which vanishes if and only if p_{θ} matches the empirical distribution (N denotes the size of the empirical sample set $\{x_i\}$ and M denotes the size of the model sample set $\{\tilde{x}_j\}$). The loss depends solely on kernel evaluations of finite samples and therefore requires no closed-form density—an essential feature in high-dimensional chaotic flows.

Parameter-update strategy. On hardware, we employ a mixed optimisation scheme. When analytic or simulator-based gradients are available (e.g. during pre-training), we use BFGS (*83*) or Adam (*84*); on a noisy quantum processor, we switch to derivative-free methods such as COBYLA (*85*) or BFGS. In both cases, a mini-batch of M circuit samples is generated per update, with 20 000 shots and M3 read-out mitigation (see Supplementary section S2.2) to suppress measurement noise on the quantum hardware.

Why the quantum generator is efficient in this setting. With 10 or 15 qubits, the Hilbert space already spans $2^{10} = 1024$ or $2^{15} = 32\,768$ computational basis states—matching the spatial resolution of our coarsest flow field. Entanglement permits the implicit encoding of multi-point velocity correlations without enumerating them explicitly; the sample-based MMD objective circumvents

intractable likelihoods and normalization constants; and the adopted optimisation schemes require only additional forward shots, avoiding ancillary qubits or deep state tomography. These properties allow the quantum generator to provide an informative, low-parameter prior that complements the classical Koopman machine learning model discussed in the main text.

In this study, we propose a hybrid quantum–classical architecture in which quantum generators are trained to learn invariant velocity distributions that characterise the long-term statistical structure of chaotic fluid systems. These learned quantum distributions serve as data-driven priors to regularize the predictions of a classical machine learning model trained on PDE dynamics. Specifically, let $p_\theta(x)$ denote the generated prior distribution and $\hat{q}(x)$ the empirical velocity distribution obtained from the prediction. We design a composite regularization strategy combining both a Kullback–Leibler (KL) divergence loss and an MMD loss:

$$\mathcal{L}_{\mathrm{KL}} = D_{\mathrm{KL}}\left(\hat{q}(x) \| p_\theta(x)\right), \tag{S4}$$

$$\mathcal{L}_{\mathrm{MMD}} = \left\| \mathbb{E}_{x\sim\hat{q}(x)}[\phi(x)] - \mathbb{E}_{x\sim p_\theta(x)}[\phi(x)] \right\|_{\mathcal{H}}^2 . \tag{S5}$$

Here, $\phi(x)$ denotes a feature mapping into a reproducing kernel Hilbert space $\mathcal{H}$, and $\hat{q}(x)$ is derived from the predicted velocity field. The KL term in equation ($A4$) captures first-order alignment between predicted and reference distributions in information-theoretic terms, while the MMD term measures higher-order statistical discrepancies under a kernel embedding. This dual-objective regularization is inspired by recent developments in physics-informed learning frameworks such as DysLIM (*30*), which have shown improved robustness in chaotic and high-dimensional regimes.

The total training objective combines the standard reconstruction loss with these quantum-informed priors:

$$\mathcal{L}_{\mathrm{total}} = \mathcal{L}_{\mathrm{recon}} + \lambda_{\mathrm{KL}}\mathcal{L}_{\mathrm{KL}} + \lambda_{\mathrm{MMD}}\mathcal{L}_{\mathrm{MMD}}, \tag{S6}$$

where $\mathcal{L}_{\mathrm{recon}} = \|\hat{u}_{t+1} - u_{t+1}\|^2$ and the hyperparameters $\lambda_{\mathrm{KL}}, \lambda_{\mathrm{MMD}}$ are selected empirically to balance fidelity and long-term physical consistency.

By anchoring machine learning model predictions to quantum-learned invariant structures, the framework improves stability during roll-out and reduces drift caused by chaotic sensitivity. The quantum modules are implemented using 10–15 qubits and 3–8 layers of parameterized rotation gates, with a total of 30–300 trainable parameters. Execution is performed on the IQM Garnet

superconducting quantum processor—a 20-qubit near-term quantum device with gate fidelities reported in (*81*) and Table S2.Circuit details, ansatz choices, and calibration parameters are provided in the next section.

These results provide an early but concrete demonstration of how quantum components may be meaningfully integrated into classical PDE solvers, offering scalable hybrid strategies even within the noise and qubit limitations of current quantum hardware.

S2 Implementation of QIML on the emulator and IQM Devices

In the QIML framework proposed in this work, the quantum component is realized using a parameterized quantum circuit as a quantum generator, as shown in Fig. S1. Its core function, learning invariant distributions from observational data, has been outlined in the preceding section. Here, we detail the implementation of this quantum model on the classical emulator and superconducting quantum hardware provided by IQM (*81*). A classical emulator is a conventional computer program that simulates the mathematical operations of an ideal, noise-free quantum device. Due to experimental resource constraints, real quantum hardware implementation was conducted only on the most challenging benchmark—the turbulent channel flow dataset.

S2.1 Quantum Circuit Architecture and Parameterization

We construct our quantum circuit using a layered structure composed of parameterized single-qubit rotations followed by entangling operations. The circuit uses Qiskit and consists of $L = 3$ to 8 alternating layers. Each layer comprises three rotation gates per qubit: $R_y(\theta_i)$, $R_z(\phi_i)$, and $R_x(\psi_i)$ which are all transpiled to local rotation gates, followed by a sequence of controlled-Z (CZ) gates between adjacent qubits. The number of qubits n ranges from 10 to 15, depending on the resolution of the discretised target distribution.

$$U(\boldsymbol{\theta}) = \prod_{\ell=1}^{L} \Big[\Big(\prod_{j=1}^{n} R_x^{(j)}(\psi_j^{(\ell)})\, R_z^{(j)}(\phi_j^{(\ell)})\, R_y^{(j)}(\theta_j^{(\ell)}) \Big) \Big(\prod_{(j,k)\in C} \mathrm{CZ}_{j,k} \Big) \Big], \tag{S7}$$

The full quantum state $|\psi_\theta\rangle = U(\theta)|0\cdots0\rangle$ is sampled N times to generate empirical frequencies $\hat{p}_\theta(x)$ used to approximate the target distribution.

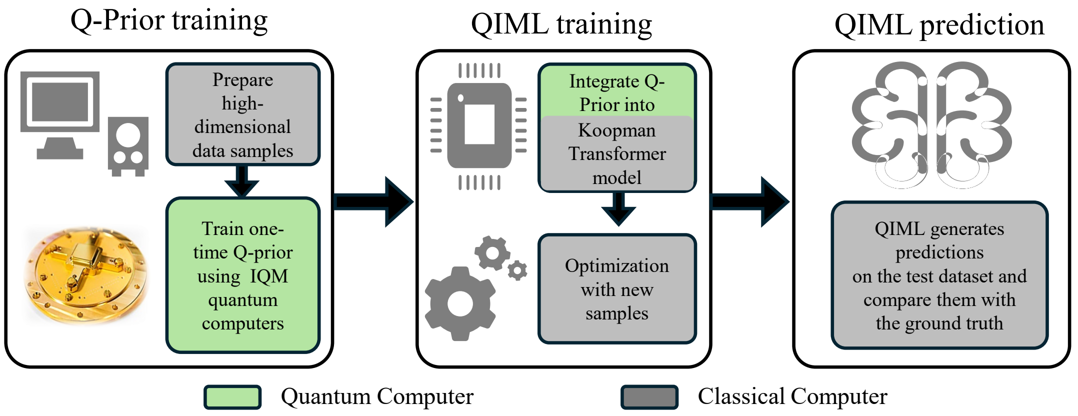

Figure S1: **Workflow of the QIML framework.** The overall process consists of three sequential stages: (left) Using high-dimensional chaotic data, a one-time offline training is performed on a quantum processor to learn a compressed Q-Prior. (middle) The pretrained and fixed Q-Prior is then integrated into the training loop of a classical machine learning model (such as a Koopman Transformer model) as a physical constraint that guides optimisation. (right) Finally, the trained QIML model performs autoregressive prediction on new data.

Notably, we do not inject explicit classical features into the quantum circuit. Instead, the quantum module acts purely as a generative module: its parameters are optimized so that the Born distribution reproduces the empirical statistics of the data. This choice eliminates costly quantum-to-classical data interfacing and highlights the expressive power of comparatively shallow circuits for modelling complex, high-dimensional distributions.

A parameterized quantum state $|\psi_\theta\rangle$ lives in a 2^n-dimensional Hilbert space, where the amplitudes $\langle x|\psi_\theta\rangle$ define a probability distribution over computational basis states. Each basis string x is mapped bijectively to a spatial grid point, so the quantum state provides an implicit embedding of the target measure into a linear feature space. Unlike explicit kernel expansions, however, this Hilbert-space representation is generated by a quantum circuit whose entangling gates can capture non-local correlations—potentially including quantum entanglement—without the need for an exponential number of classical parameters.

This construction allows for efficient sampling and generalization from complex distributions. Classical networks often require thousands of parameters to represent distributions with long-range

correlations or multi-modal structure. In contrast, our Q-Prior uses fewer parameters and leverages the quantum state's exponential support to encode richer structure in the generated samples. This trade-off between circuit expressivity and parameter efficiency is particularly valuable under the constraints of quantum hardware.

S2.2 Implementation of QIML

We implemented and validated the classical machine learning and quantum emulator on BEAST GPU cluster from Leibniz Supercomputing Centre and the quantum generator on superconducting quantum hardware provided by IQM, mainly using the 20-qubit *Garnet* chip. A subset of 10-15 qubits was selected based on individual coherence performance and gate error metrics. The above circuit is compiled to superconducting hardware using IQM's transpiler stack. Transpilation includes single- and two-qubit gate fusion, qubit remapping, and hardware-specific gate decompositions. A representative transpiled circuit for the 10-qubit Garnet chip is shown in Supplementary Fig. S2, illustrating the translation of $R_y/R_z/R_x$ blocks into native $R(\theta, \phi)$ rotations with optimized layout and connectivity. The total number of parameters is approximately bounded by 300, as each qubit hosts 2–3 rotation gates per layer (each with a trainable parameter), and the circuit involves up to 10 qubits and 10 layers.

Each quantum circuit is executed with $N = 20{,}000$ measurement shots on the emulator and quantum devices, yielding samples $\{x_i\}_{i=1}^{N}$ distributed according to the Born rule, i.e., with probabilities $|\langle x_i|\psi_\theta\rangle|^2$. These are post-processed into traditional histograms and compared to the empirical fluid velocity distribution via KL divergence, MMD loss, and peak structure preservation losses. Sampling outcomes typically yield 8000–20,000 distinct outcomes per shot batch, corresponding to 10–15-bit strings depending on circuit size. All remaining traditional computation, including forward simulation and loss evaluation, was executed on NVIDIA A100 GPUs. To provide context on the practical feasibility of our approach, Table S1 compares the resource budget of the QIML framework with that of established quantum algorithms, such as VQE and HHL. It highlights that the QIML framework has significantly lower requirements for circuit depth and measurement overhead.

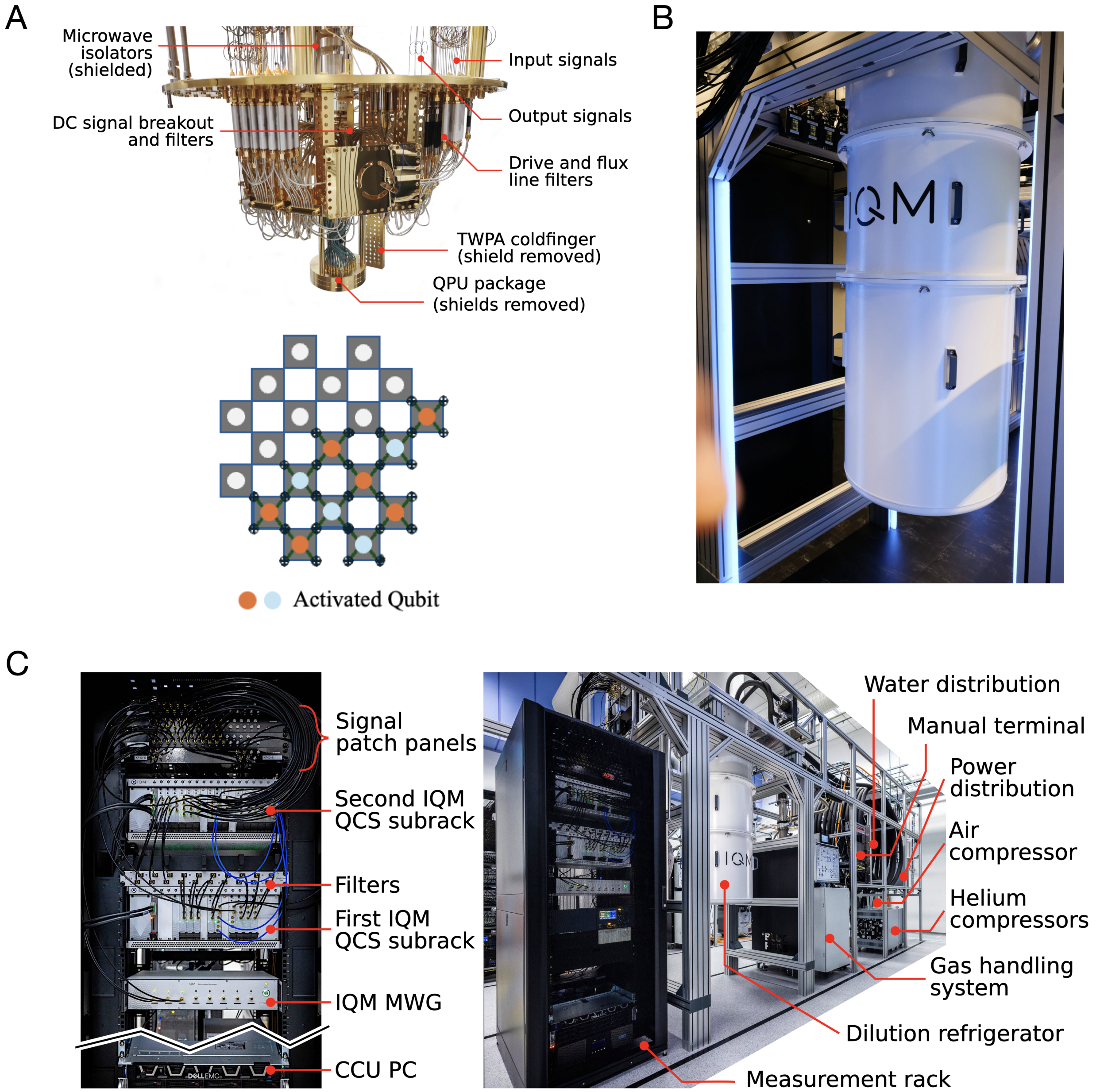

Figure S2: **Representative images of the IQM Radiance 20 quantum computer (Garnet).** Copyright permission from IQM. A. Cryogenic components inside the host dilution refrigerator. 10 qubits are used of the 20-qubit IQM computer. B. IQM's quantum computer is housed within an ultra-high vacuum cryostat during operation, maintaining the ultra-low temperatures required for superconducting qubit performance. c. Photograph of the IQM system without covers.

S2.3 Optimisation Strategy and Training Protocol

The training for the Kuramoto-Sivashinsky and Kolmogorov flow systems was conducted on a quantum circuit emulator, which is a classical simulation of an ideal quantum device supported by the PennyLane and Qiskit libraries, optimized by Adam. Given its higher complexity and the failure of classical models to learn its dynamics, the TCF system was used for validation on real quantum hardware, accessed via Qiskit and the IQM-Qiskit provider. As shown in Fig. S3, for the training on the hardware, our optimisation of quantum generator parameters was carried out using several traditional algorithms, with L-BFGS yielding the most stable convergence under hardware noise. Across all simulations and experiments, and depending on dataset complexity and resolution, training epochs varied between 400 and 500. The number of learnable parameters, corresponding to the angles of the parameterized rotation gates within the quantum circuit as shown in Fig. 9, ranged from 120 to 300. For the specific parameter count for each system, we refer the reader to Table S3 and Table S4 in our discussion on parameter efficiency and memory advantage. Target distributions were generated by binning velocity fields from turbulent and Kolmogorov flow datasets into one-dimensional histograms with support ranging from $2^{10} = 1024$ to $2^{15} = 32,768$ bins.

S2.4 Sensitivity of Hardware Quantum Generator to Qubit Number and Circuit Depth

To better understand the scalability limitations of Q-priors on real quantum hardware, we conducted an ablation study by varying both the number of active qubits (from 4 to 15) and the circuit depth (from 2 to 12 layers) on the IQM superconducting processors. As shown on the IQM official website, increasing either the qubit count or the number of layers leads to a marked rise in total circuit error, which in turn degrades the quality of the sampled distributions—particularly their ability to reproduce accurate velocity statistics.

This degradation is primarily due to the accumulation of readout noise and gate errors, which increase non-linearly with circuit size. In contrast to noiseless simulators or emulators, real hardware suffers from decoherence, sampling noise, and hardware-specific imperfections that compound as more gates and qubits are used. These effects place practical constraints on how expressive a quantum circuit can be without error correction. Such challenges are hallmarks of the present NISQ

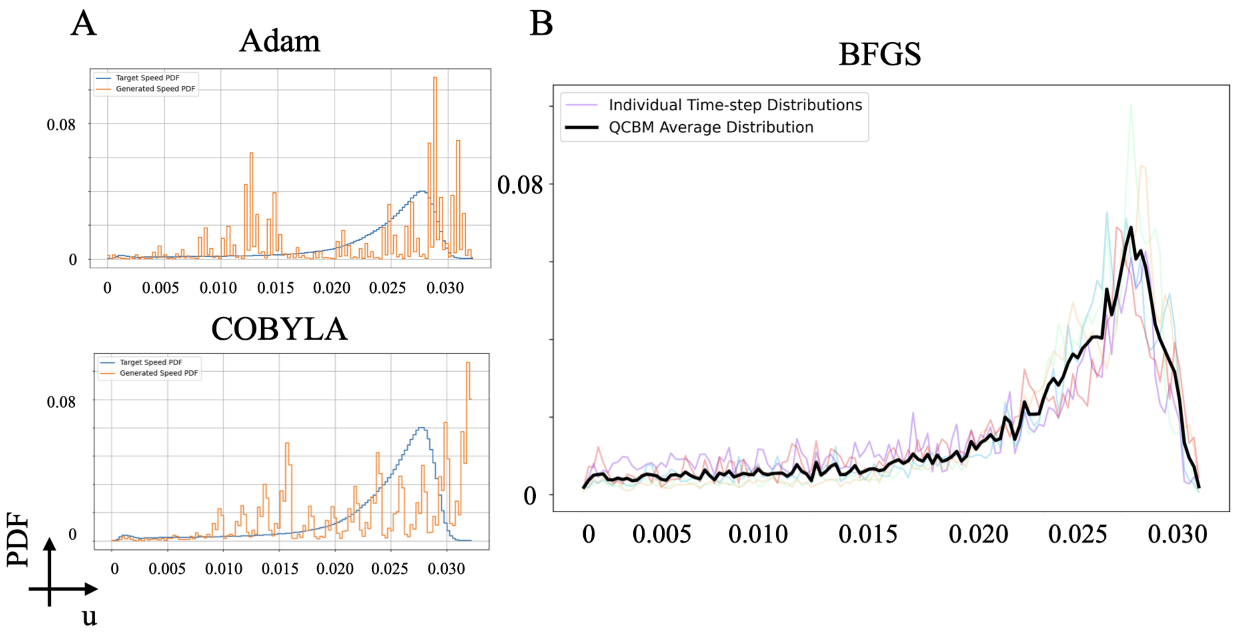

Figure S3: **Quantum generator results obtained using different optimisation methods.** A. Training with Adam and COBYLA optimizers results in poor convergence and noisy distributions. B. Applying a single-shot L-BFGS optimizer in combination with the M3 measurement mitigation technique yields significantly improved performance.

era, where the accumulation of errors fundamentally limits computational power.

To address these limitations, we apply hardware-aware strategies including targeted chip and qubit selection, suitable circuit depth, and error mitigation, which are detailed in the following section.

S2.5 Chip Selection, Error Mitigation and Robustness Analysis

As shown on IQM's official website and Fig. S4, our initial experiments using IQM's *Sirius* chip yielded not good convergence due to fidelity limitations, two-bit gate connection limitations due to hardware topology, and the inherent sensitivity of high-resolution distributions to noise. The technical specifications and performance metrics, including coherence times and gate fidelities, for the Sirius and Garnet quantum processors are summarized in Table S2. To mitigate these issues, we first migrated our algorithm to the *Garnet* high-fidelity architecture and reduced histogram binning granularity from 1024 to 256. To mitigate the effect of readout errors during quantum sampling, we implemented a post-processing strategy based on the matrix-free measurement mitigation (`M3`) protocol (*82*) shown in Fig. 9. This technique calibrates the readout noise by learning a probabilistic response model from the device's native measurement behaviour. Then it applies Bayesian corrections to raw bitstring outputs without explicitly inverting a response matrix, thereby preserving numerical stability at scale. The use of `M3` proved particularly valuable given the multi-qubit readout complexity and the high measurement resolution required to fit fine-grained distributional features.

Additionally, in the present work, practical experiments were conducted at a reduced resolution (see last paragraph). Scaling the QIML framework to a high-resolution geophysical grid of 1024×1024 points (approx. 10^6 degrees of freedom) requires encoding a Hilbert space of dimension 2^{20}. Assuming a compact generative ansatz, this necessitates a minimum of $n \approx 20$ logical qubits for spatial addressing, plus auxiliary qubits for feature channels, placing the requirement in the range of 30-50 qubits. Regarding coherence, capturing the multi-scale correlations of such a large system likely requires increased circuit depth, estimated at $D \sim 50 - 100$ layers. To maintain a meaningful signal-to-noise ratio without full error correction, the two-qubit gate fidelity would need to exceed 99.9%, a target approachable by next-generation superconducting or trapped-ion

processors combined with advanced error mitigation strategies.

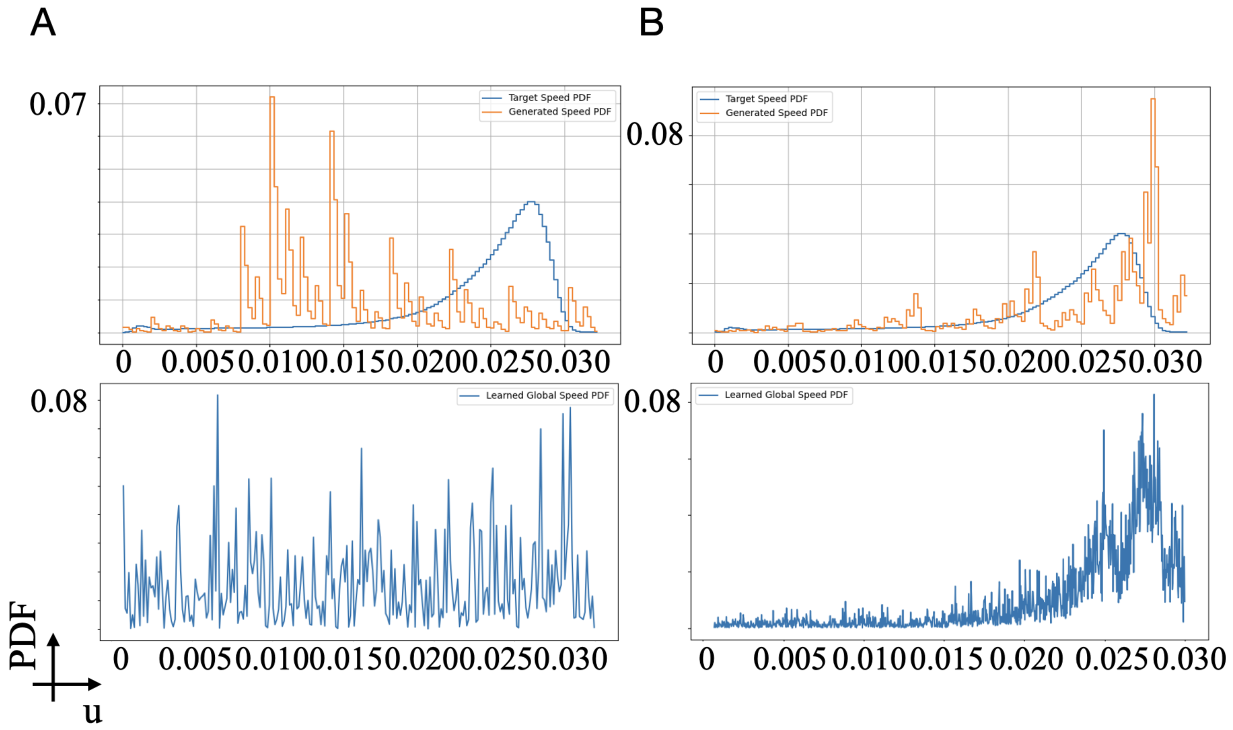

Figure S4: **Quantum generator results obtained on different IQM quantum devices.** A. Training on IQM-Sirius results in poorer distributions. B. Quantum Generator achieves better results on IQM-Garnet.

In addition to `M3`, we employed a combination of large-sample averaging and outlier rejection to suppress noise further. One key feature of our framework is the one-time, offline training of the Q-prior. The quantum generator is trained for a total of 50 epochs. Once this initial training process is complete, the quantum circuit is no longer needed for the main training loop. Within each of the 50 training epochs, each quantum circuit was executed with up to 20,000 shots, and the resulting histograms were averaged across repeated trials to stabilize statistical fluctuations. Outlier detection routines were applied to eliminate anomalous measurement rounds—specifically, instances where a disproportionate number of outcomes collapsed into trivial all-zero or all-one configurations. These spurious distributions, often symptomatic of transient decoherence or control drift, were discarded from the final ensemble to avoid biasing the learned quantum distribution. Together, these techniques enabled robust extraction of high-dimensional statistical structure from quantum circuits

on quantum hardware. Circuit transpilation was performed via IQM's native compiler stack, which supports qubit mapping, single-gate merging, and readout-aware optimisation. These improvements allowed us to stabilize the training loss and achieve consistent sampling performance across multiple runs. We tested numerous qubit configurations on both 10-qubit and 15-qubit hardware layouts. Results show that expressive distributions can be reliably approximated using $\sim$120 parameters, with deeper circuits providing better capture of multimodal or heavy-tailed distributions. The absence of input encoding enables this model to generalize across different datasets (e.g., Kolmogorov flow and shear-driven turbulence) by retraining only on the output distribution. We observed consistent convergence behaviours across independent runs, with less than 5% variance in MMD divergence across 5 seeds.

Furthermore, we found that higher-resolution distributions exhibit increased robustness against circuit-level noise, while low-resolution cases suffer from distributional collapse and mode imbalance. The experimental evidence suggests that quantum circuits are particularly well-suited to modelling high-dimensional dynamical systems. These findings support the feasibility of embedding small-scale quantum devices within traditional learning pipelines. The successful deployment of quantum generators on real quantum hardware, together with GPU-based optimisation and software differentiation, illustrates a viable hybrid computing paradigm for physics-informed machine learning.

S3 Kuramoto–Sivashinsky Equation

The Kuramoto-Sivashinsky (KS) equation is a fourth-order nonlinear partial differential equation that models spatiotemporal instabilities in a range of physical systems (*102, 103*). It is especially important in the study of pattern formation and chaos. The governing equation is described as follows.

S3.1 Governing Equation

In one spatial dimension, the KS equation is written as:

$$\frac{\partial u}{\partial t} + u\frac{\partial u}{\partial x} + \frac{\partial^2 u}{\partial x^2} + \nu\frac{\partial^4 u}{\partial x^4} = 0, \tag{S8}$$

where $u(x,t)$ is a scalar field, x is the spatial coordinate, t is time, and ν is a positive parameter controlling the strength of the fourth-order dissipation term. The term $\frac{\partial u}{\partial t}$ describes the temporal evolution of the field. The nonlinear term $u\frac{\partial u}{\partial x}$ represents convective transport and introduces non-linearity into the system. The second derivative term $\frac{\partial^2 u}{\partial x^2}$ acts as a linear destabilizing mechanism, similar to anti-diffusion, amplifying short-wavelength perturbations. The fourth derivative term $\nu\frac{\partial^4 u}{\partial x^4}$ provides a stabilizing effect by damping high-frequency modes, thereby preventing blow-up and enabling bounded chaotic behaviour.

S3.2 Boundary Conditions

In this study, the KS equation is performed under periodic boundary conditions of the form: $u(x+L,t) = u(x,t)$, where L is the spatial period of the domain. These conditions reflect the translational symmetry of many physical systems and simplify the analysis of chaotic dynamics.

S3.3 Data Source

In the first application, we performed KS equation dataset with the help of CFD jax community code (*59*). The dataset has also been used and validated in Ref. (*30*).

S4 Kolmogorov Flow Governing Equation

The Kolmogorov flow is governed by the incompressible Navier-Stokes (NS) equations with a sinusoidal forcing term. In this paper, we apply a 2D Kolmogorov flow as an example to examine our QIML framework. Below, we will describe our system in 2D NS equations with forcing.

S4.1 Navier-Stokes Equations with Forcing

The flow is described by the incompressible Navier-Stokes equations with an external forcing term, which is denoted as

$$\frac{\partial \mathbf{u}}{\partial t} + (\mathbf{u} \cdot \nabla)\mathbf{u} = -\nabla p + \nu \nabla^2 \mathbf{u} + \mathbf{F}, \tag{S9}$$

$$\nabla \cdot \mathbf{u} = 0, \tag{S10}$$

where $\mathbf{u} = (u(x, y, t), v(x, y, t))$ is the velocity vector field of the fluid, $p(x, y, t)$ is the scalar pressure field, ν is the kinematic viscosity, and $\mathbf{F}$ represents the external body force applied to the fluid.

S4.2 Kolmogorov Forcing

In the Kolmogorov setup, the force is applied only in the x-direction and varies sinusoidally in the y-direction. The forcing term is defined as:

$$\mathbf{F} = (F_0 \sin(ky), 0), \tag{S11}$$

where F_0 is the amplitude of the forcing and k is the wavenumber determining the periodicity in the y-direction.

S4.3 Data Source

In the second application, we utilized the high-fidelity Kolmogorov dataset derived from (*104*), which offers a comprehensive and statistically rich representation of turbulent flow fields. This dataset was critical for evaluating the robustness and generalization of our proposed model, particularly under complex conditions characterized by a high Reynolds number of $Re = 1000$. It comprises 40 trajectories, each containing 320 temporal snapshots, with a spatial resolution of 256×256 grid points. To establish a high-fidelity ground truth, all numerical simulations for data generation were performed using double-precision (FP64) floating-point arithmetic. For the machine learning stage, however, our QIML framework operates entirely in single-precision (FP32) during both training and inference, a standard practice to balance computational efficiency and numerical stability. Consequently, while the models are trained on FP64 data, their predictive outputs are themselves of FP32 precision. The extent to which training the QIML framework in double-precision would alter these predictive outcomes remains a subject for future study.

S5 LBM-based 3D turbulent channel flow simulation

S5.1 The Lattice Boltzmann Method

The Lattice Boltzmann Method is known as an alternative computational fluid dynamics (CFD) framework that models the evolution of single particle distribution functions at the kinetic-level. It is based on the discrete form of the Boltzmann equation and operates on a lattice grid in space and time. The governing equation for the probability distribution function, or populations $\mathbf{f}$, located at position $\mathbf{x}$ at time t, accounting for both collisions and external forces, is given by:

$$\mathbf{f}(\mathbf{x}+\mathbf{c}_i\Delta t, t+\Delta t) = \mathbf{f}(\mathbf{x}, t) + \Omega\left(\mathbf{f}(\mathbf{x}, t)\right) + \mathbf{F}(\mathbf{x}, t), \tag{S12}$$

where $\mathbf{c}_i$ are the discrete lattice velocities, Δt is the simulation time step which is set to unity, Ω is the collision operator for the probability distribution function. $\mathbf{F}$ is the external volume force.

The LBE has gained popularity due to its simplicity, ease of implementation on parallel architectures, and its ability to naturally handle complex boundary conditions. Macroscopic fluid quantities such as density and velocity are obtained by taking moments of the distribution function. Specifically, the fluid density ρ and momentum density $\rho\mathbf{u}$ are computed as follows:

$$\rho(\mathbf{x}, t) = \sum_{i=0}^{Q-1} f_i(\mathbf{x}, t), \tag{S13}$$

$$\rho(\mathbf{x}, t)\mathbf{u}(\mathbf{x}, t) = \sum_{i=0}^{Q-1} f_i(\mathbf{x}, t)\mathbf{c}_i, \tag{S14}$$

where $f_i(\mathbf{x}, t)$ is the particle distribution function in the i-th discrete velocity direction at position $\mathbf{x}$ and time t.

S5.2 Bhatnagar–Gross–Krook Collision Kernel

We define BGK collision kernel Ω as follow:

$$\Omega\left(\mathbf{f}(\mathbf{x}, t)\right) = -\frac{1}{\tau}(\mathbf{f}(\mathbf{x}, t) - \mathbf{f}^{\text{eq}}(\mathbf{x}, t)), \tag{S15}$$

where $\mathbf{f}^{\text{eq}}(\mathbf{x}, t)$ denoted as the equilibrium distribution function, τ is correlated with the kinematic viscosity ν:

$$\nu = c_s^2\left(\tau - \frac{1}{2}\right)\Delta t_{coll}, \tag{S16}$$

where Δt_{coll} is set to identity in the simulation.

S5.3 Smagorinsky Subgrid-Scale Modelling

In this part, we summarize the lattice-Boltzmann-based Smagorinsky Subgrid Scale (SGS) LES techniques. Within the LBM framework, the effective viscosity ν_{eff} (*105–107*) is modeled as the sum of the molecular viscosity, ν_0, and the turbulent viscosity, ν_t:

$$\nu_{\text{eff}} = \nu_0 + \nu_t, \qquad \nu_t = C_{\text{smag}}\Delta^2 \left|\bar{\mathbf{S}}\right|, \tag{S17}$$

where $\left|\bar{\mathbf{S}}\right|$ is the filtered strain rate tensor, C_{smag} is the Smagorinsky constant, Δ represents the filter size, which is set to 1 LBU. The Smagorinsky constant for this study is set to $C_{\text{smag}} = 0.01$.

S5.4 Simulation Set up

In this study, the computational domain for the turbulent channel flow simulation is defined with dimensions $L_x \times L_y \times L_z = 1024 \times 192 \times 192$, where x, y, and z denote the streamwise, vertical, and spanwise directions, respectively. The friction Reynolds number is set to $Re_\tau = 180$ which is equivalent to $Re = 3250$. Periodic boundary conditions are applied in the streamwise and spanwise directions, while the vertical direction is governed by no-slip boundary conditions (*108*). This configuration distinguishes our dataset from existing studies that primarily rely on 2D turbulent cases, as our dataset is based on fully three-dimensional simulations. The fully developed 3D turbulent channel flow simulation follows the configuration outlined in reference (*62*). The simulation begins from an initial zero-velocity field, with a square block of size $20 \times 20 \times 100$ grid points positioned at $x = 192$. A volumetric force is applied uniformly across the domain to drive the flow. The simulation is run for 50 domain-through times to establish initial flow characteristics. After this initial phase, the block is removed, and the simulation is continued for an additional 50 domain-through times, allowing the flow to fully develop into a turbulent state. Data sampling begins after this stage, focusing on the 2D cross-section at $x = 512$. Data is collected over a further 100 turnover times, ensuring that the samples represent fully developed turbulence. The simulation timestep is set to $\Delta t = 0.02\,\text{s}$, and it operates in dimensionless units (800 timesteps), as is typical for LBM simulations. The detailed transformation from dimensionless units to physical units can be found

in section S6. To ensure robustness and generality, we conducted three independent 3D turbulent channel flow simulations under these conditions, generating a comprehensive dataset for analysis.

S5.5 Dataset Periodic Turbulent Channel Flow Validation

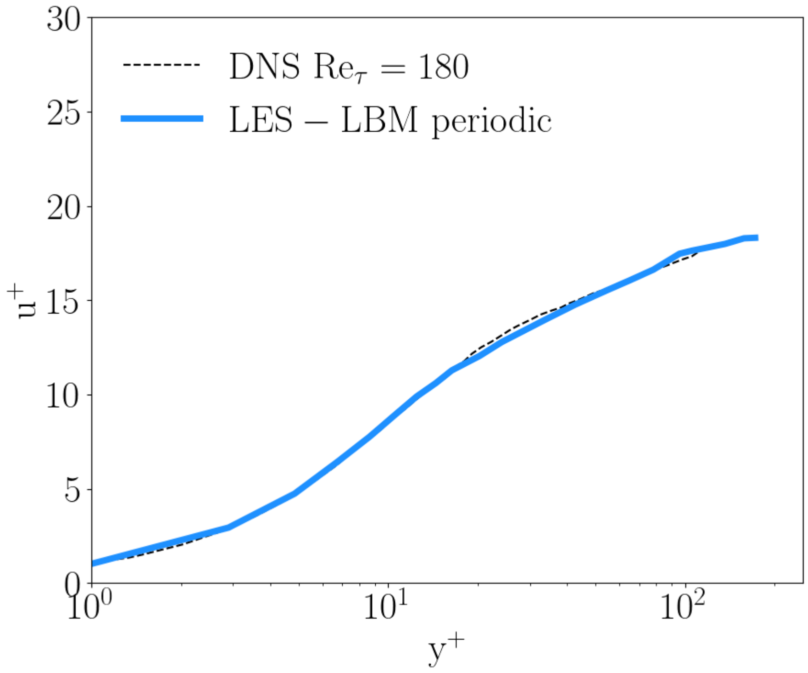

Figure S5: **Comparison of dimensionless mean streamwise velocity profiles in wall units (u^+ vs. y^+) between the LES–LBM simulation with periodic boundary conditions and DNS data at Re$_\tau$ = 180.** The LES–LBM result (solid blue) closely follows the reference DNS profile (dashed black), validating the accuracy of the LBM in capturing the near-wall behaviour and log-layer scaling of turbulent channel flow.

Fig. S5 presents a comparison between the streamwise mean velocity profile obtained from our large eddy simulation using the lattice Boltzmann method (LES–LBM) and benchmark direct numerical simulation (DNS) data at a friction Reynolds number of Reτ = 180. The velocity is normalized in wall units, where $u^+ = u/u_\tau$ and $y^+ = yu_\tau/\nu$. The LES–LBM result shows good agreement with the DNS reference across both the viscous sublayer and the logarithmic region, indicating that the model successfully captures key features of wall-bounded turbulence under periodic boundary conditions.

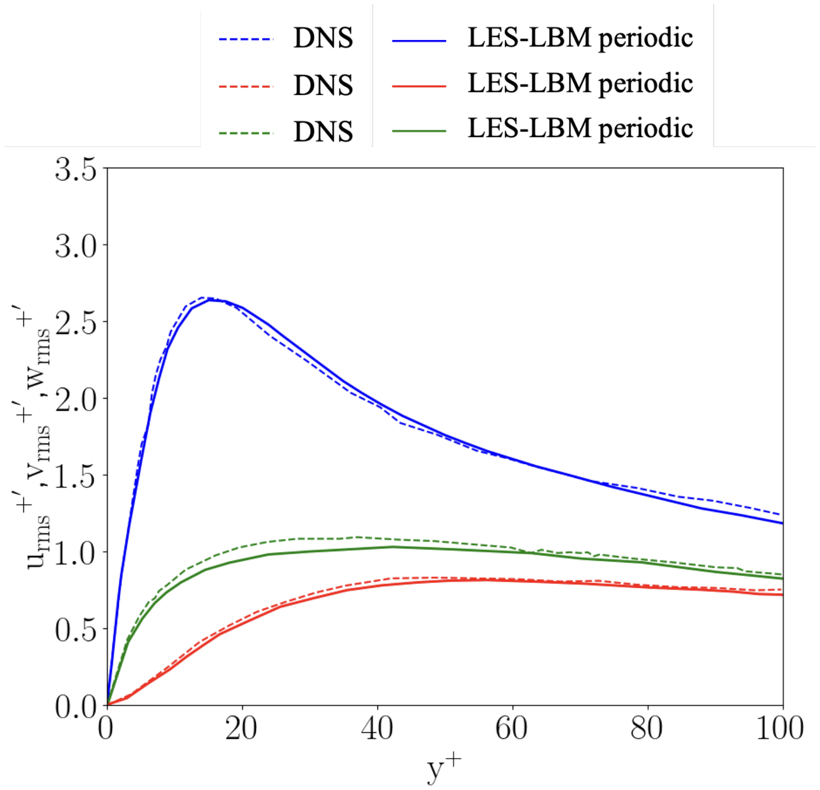

Figure S6: **Comparison of root-mean-square (RMS) velocity fluctuations in dimensionless wall units between LES–LBM simulations with periodic boundary conditions and DNS data at Re$_\tau$ = 180.** The streamwise (blue), wall-normal (red), and spanwise (green) RMS components are shown. The LES–LBM results (solid lines) closely match the DNS reference (dashed lines), particularly in the near-wall region, capturing the peak in $u^{+'}_{\rm rms}$ and maintaining the correct anisotropy among components.

S6 shows the comparison of RMS velocity fluctuations between our LES–LBM simulations and DNS data at $\mathrm{Re}_\tau = 180$. The LES–LBM results (solid lines) exhibit good agreement with DNS (dashed lines), particularly in reproducing the characteristic peak in $u^{+'}_{\mathrm{rms}}$ near $y^+ \approx 15$ and capturing the general anisotropic distribution of turbulent fluctuations across the channel height.

S5.6 The Multiple-Relaxation Time Lattice Boltzmann Moment Space Transformation Matrix

Let's revisit the evolution equation for the distribution functions expressed as:

$$\mathbf{f}(\mathbf{x} + \mathbf{c}_i \Delta t, t + \Delta t) = \mathbf{f}(\mathbf{x}, t) - \mathbf{M}^{-1}\mathbf{S}\mathbf{M}\left[\mathbf{f}(\mathbf{x}, t) - \mathbf{f}^{\,\mathrm{eq}}(\mathbf{x}, t)\right] + \mathbf{F}(\mathbf{x}, t)\Delta t, \tag{S18}$$

where $\mathbf{M}$ denotes the moment space transformation matrix for the multiple relaxation time (MRT) collision kernel (*109*). The explicit matrix is shown in the supplementary Table S5.

S6 Unit Conversion between LBM Units and Physical Units

In this subsection, we demonstrate the unit conversion between LBU and physical units in both velocity and distance. Within the LBM simulation, we can obtain the LBM velocity $\mathbf{u}_{\mathrm{LB}}(\mathbf{x}, t)$ at location $\mathbf{x}$. The physical unit of the velocity, $\mathbf{u}_{\mathrm{phys}}(\mathbf{x}, t)$, can be written as

$$u_{\mathrm{phys}}(\mathbf{x}, t) = u_{\mathrm{LB}}(\mathbf{x}, t)\frac{c_x}{c_t}, \tag{S19}$$

where c_x and c_t are the conversion factors from the lattice Boltzmann simulation to the physical system, which is defined as

$$c_x = \frac{L_{\mathrm{phys}}}{L_{\mathrm{LB}}}, \qquad c_t = \frac{t_{\mathrm{phys}}}{t_{\mathrm{LB}}}, \tag{S20}$$

where L_{phys} and t_{phys} represent the space and time units from the physical system, while L_{LB} and t_{LB} are the space and time lattice Boltzmann units from the lattice Boltzmann simulation. Accordingly, we can also obtained the physical distance y_{phys} from the LBM length scale:

$$y_{\mathrm{phys}} = y_{\mathrm{LB}} c_x. \tag{S21}$$

For example, in Eq. S20, L_{phys} may denote the height of the channel as $L_{\mathrm{phys}} = 2\mathrm{m}$ so that $L_{\mathrm{LB}} = 20$ LBU. Then, $c_x = 0.1\mathrm{m}$. Similar calculations can be performed to determine the time scale conversion factor c_t.

S7 Neural Networks Architecture Details

S7.1 Quantum Machine Learning Model Architecture

The quantum machine learning model consists of parameterized quantum circuits trained to approximate target Q-Priors via a quantum generator; the optimisation is performed traditionally through gradient-based updates, as detailed in Supplementary S1 and illustrated in Fig. 1 of the main text.

In our QIML architecture, quantum and traditional computations are optimized independently but operate in a tightly coupled feedback loop. The outputs of traditional modules dynamically guide quantum circuit objectives, establishing a synergistic interaction that underscores the relevance and practicality of hybrid quantum–traditional integration.

S7.2 Model Architecture for 2D chaotic flows

The Koopman-based model begins with a patch embedding layer implemented using a single 2D convolutional layer. This layer transforms the input from 1 channel to 32 channels while maintaining a spatial resolution of $(64, 64)$. No activation function is specified for this layer.

Following the embedding, the encoder consists of three transformer blocks. The first block contains 2 layers and maps features from 32 channels to 64 channels, reducing the spatial dimensions to $(32, 32)$, with ReLU activations. The second block has 3 layers and increases the channels from 64 to 128 while downsampling to $(8, 8)$, again using ReLU activations. The third block also comprises 3 layers, maintaining 128 channels and compressing the spatial dimensions to $(2, 2)$, with ReLU activation applied.

After encoding, the representation is passed through a fully connected layer that operates on a flattened feature vector of size $128 \times 2 \times 2$. The activation function is not specified for this operator. Next, the model includes a fully connected layer applied to the same flattened $128 \times 2 \times 2$ feature vector, without a specified activation.

A third component, the backward component, is similarly implemented using a fully connected layer acting on the $128 \times 2 \times 2$ vector, with no activation mentioned.

The decoder mirrors the encoder architecture in reverse. The first decoder block uses 3 transformer layers to maintain 128 channels and upsample to $(8, 8)$ with ReLU activation. The second

block also has 3 layers, reducing the channels from 128 to 64 and increasing the spatial resolution to $(32, 32)$, again with ReLU. The third block consists of 2 layers, mapping 64 channels to 32 with output size $(64, 64)$ and ReLU activations. Finally, a single 2D convolutional layer reduces the channels from 32 back to 1 while preserving the $(64, 64)$ spatial resolution, with no activation function specified.

Parameter comparison across different models (QIML, Koopman, FNO and MNO) can be found in Table S3.

S7.3 Fourier Neural Operator Architecture

The Fourier Neural Operator is a neural network architecture designed to learn mappings between function spaces, with a particular focus on solving partial differential equations and modelling spatial-temporal systems. Unlike traditional convolutional networks, it operates in the frequency domain, enabling efficient learning of long-range dependencies.

The processing pipeline in this paper consists of three main stages:

Lifting, which maps the input tensor from $C_{\text{in}} = 1$ to $C_{\text{hidden}} = 128$ channels using a 1×1 convolution:

$$\mathbf{X}_1 = \text{Conv}_{1\times1}(\mathbf{X}_0)\,, \tag{S22}$$

where $\mathbf{X}_0 \in \mathbb{R}^{B,1,H,W}$ and $\mathbf{X_1} \in \mathbb{R}^{B,128,H,W}$.

Four Fourier layers, each of which is formed by spectral convolutional blocks with residual connections. The spectral convolution block first applies a 2D Fast Fourier Transform to the input tensor along its spatial dimensions. Then, a mode truncation step retains only the lowest $n_{\text{mode}} = (32, 32)$ frequency components. These retained modes are acted upon by complex-valued spectral weights $\mathbf{W}_{\text{spec}} \in \mathbb{C}^{C_{\text{out}}\times C_{\text{in}}\times 32\times 32}$. To reduce storage and computational cost, these spectral weights are factorized using Tucker factorization as:

$$\mathbf{W}_{\text{spec}} \approx \mathbf{U}_{\text{out}} \times \mathbf{G} \times \mathbf{U}_{\text{in}}\,, \tag{S23}$$

where $\mathbf{U}_{\text{in}} \in \mathbb{R}^{C_{\text{in}}\times r_{\text{in}}}$, $\mathbf{U}_{\text{out}} \in \mathbb{R}^{C_{\text{out}}\times r_{\text{out}}}$ and $\mathbf{G} \in \mathbb{C}^{r_{\text{in}}\times r_{\text{out}}\times 32\times 32}$. The modified spectrum is then transformed back to the spatial domain via an inverse FFT. In parallel, a pointwise convolution path applies a 1×1 convolution to the original input tensor to retain local information. Finally, a

residual connection combines these paths with the original input, followed by a GELU non-linear activation function:

$$\mathbf{X}_{\mathcal{L}+1} = \sigma(\text{Spectral}(\mathbf{X}_{\mathcal{L}}) + \text{Pointwise}(\mathbf{X}_{\mathcal{L}}) + \mathbf{X}_{\mathcal{L}}) . \tag{S24}$$

Projection, the final stage, which reduces the hidden representation from $C_{\text{hidden}} = 128$ to $C_{\text{out}} = 1$ channels using a 2-layer 1×1 convolutional MLP:

$$\mathbf{Y} = \text{Conv}_{1\times1}(\sigma(\text{Conv}_{1\times1}(\mathbf{X}_{\mathcal{L}}))) , \tag{S25}$$

with intermediate channels set to $r_{\text{proj}} \times C_{\text{hidden}} = 1 \times 128 = 128$.

See Table S3 for the total parameters of the FNO model.

S7.4 Markov Neural Operator Architecture

The Markov Neural Operator is a framework to learn discretization-independent mappings between function spaces. In this approach, the learned operator is expressed as a finite composition of Markov kernel layers, each representing a local operator that evolves the state over a discrete step in "operator depth."

The architecture setting in this paper consists of three main stages:

Lifting, where a 1×1 convolution projects the input tensor from $C_{\text{in}} = 1$ to $C_{\text{hidden}} = 128$:

$$\mathbf{X}_1 = \text{Conv}_{1\times1}(\mathbf{X}_0) , \tag{S26}$$

where $\mathbf{X}_0 \in \mathbb{R}^{B,1,H,W}$ and $\mathbf{X}_1 \in \mathbb{R}^{B,128,H,W}$.

Single Markov kernel block, which consists of four sequential Markov kernel layers. Each layer is implemented via a spectral convolution in Fourier space combined with local pointwise updates, following a similar setting as the FNO architecture described previously. A residual connection combines these paths, but the non-linear activation function used is SELU. This composition ensures that each layer satisfies the Markov property, where the updated state depends only on the previous state.

Projection, the final stage, which reduces the hidden representation from $C_{\text{hidden}} = 128$ back to $C_{\text{out}} = 1$ channels.

Another key part of the MNO is its explicit stability features, which are realized by incorporating dissipative target mappings and partition of unity post-processing in the training loop. First, training inputs are drawn using spherical shell sampling:

$$\mathbf{x} \sim \mathcal{U}(\mathcal{S}(R_{\text{inner}}, R_{\text{outer}})) \tag{S27}$$

where $\mathcal{S}(r_1, r_2) = \{\mathbf{v} \in \mathbb{R}^n \mid r_1 \leq \|\mathbf{v}\|_2 \leq r_2\}$. This ensures diverse energy levels are present in the training set. Second, a baseline dissipative target mapping is defined as:

$$\mathcal{D}(\mathbf{x}) = s \cdot \mathbf{x}, \quad 0 < s < 1 \tag{S28}$$

where we use $s = 0.8$. This map serves as a stable fallback for states outside the data manifold. Finally, a partition of unity correction blends the learned mapping f_θ with the dissipative baseline $\mathcal{D}$ using a smooth partition of unity function ρ:

$$\hat{\mathbf{y}}(\mathbf{x}) = \rho(\|\mathbf{x}\|) \cdot f_\theta(\mathbf{x}) + (1 - \rho(\|\mathbf{x}\|)) \cdot \mathcal{D}(\mathbf{x}) \tag{S29}$$

with ρ defined as a sigmoid function $\rho = (1 + e^{s(r-c)})^{-1}$, where c is the shift and s controls the transition steepness. This ensures a high-fidelity learned output when $\rho \approx 1$ (inside the training region) and a stable dissipative fallback when $\rho \approx 0$ (outside the training region).

See Table S3 for the total parameters of the MNO model.

S7.5 Variational Autoencoder Baseline for the Classical Prior

To provide a classical generative baseline aligned with the Q-Prior, we consider a standard VAE architecture to construct a C-Prior. The VAE is employed purely as an unconditional generative model for learning the invariant marginal distribution of local velocity magnitudes, rather than for reconstructing full spatial fields or performing conditional prediction.

The VAE architecture follows a canonical encoder–decoder design operating on scalar inputs (*63*). The encoder maps a one-dimensional velocity sample $v \in \mathbb{R}$ to a latent representation $z \in \mathbb{R}^{d_z}$ via a multilayer perceptron, producing the parameters of a Gaussian variational posterior $q_\phi(z|v) = \mathcal{N}(\mu(v), \sigma^2(v))$. The decoder is a symmetric MLP that maps samples drawn from the latent prior $p(z) = \mathcal{N}(0, I)$ back to the velocity space. Training is performed by maximising the

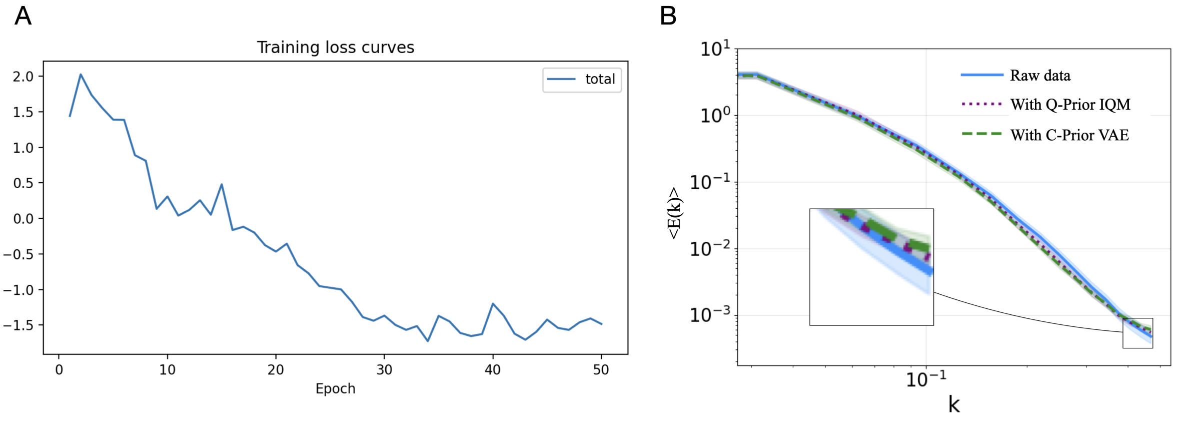

Figure S7: **Training behaviour and spectral comparison for Q-Prior and C-Prior in turbulent channel flow.** A. Representative training loss curve for the classical generative prior, illustrating stable convergence over optimisation epochs. B. Time-averaged kinetic energy spectra E(k) for the turbulent channel inflow, comparing the ground-truth data with predictions obtained using the Q-Prior (300 parameters) and the VAE-based C-Prior (more than 10^5 parameters). While both priors reproduce the large-scale spectral behaviour, deviations become apparent in the middle and high-wavenumber regime, where the Q-Prior more faithfully preserves the energy content at small scales. Insets highlight the differences in the spectral tail.

standard evidence lower bound, consisting of a reconstruction term and a Kullback–Leibler regularisation term. To ensure a fair and controlled comparison, the learning procedure for the C-Prior is designed to be statistically identical to that of the Q-Prior. Specifically, the VAE is trained on the same dataset and target quantity. Consequently, the only distinction between the C-Prior and Q-Prior lies in the representational mechanism, classical latent variables versus a parameterized quantum circuit, rather than in the data, objective, or training protocol.

Using a sufficiently high-capacity VAE (more than 10^5 trainable parameters), the resulting C-Prior is able to reproduce the coarse-grained energy distribution and large-scale statistics at a level comparable to the Q-Prior (see Fig. 7). However, beyond intermediate prediction windows, the corresponding CIML rollouts exhibit a tendency toward dynamical saturation, with the predicted flow becoming progressively less variable in time and approaching a quasi-stationary configuration. Consistent with this observation, Fig. S7 shows modest but systematic deviations in the high-wavenumber regime, where the classical prior slightly underestimates the tail of the energy spectrum relative to QIML. While these differences are small at the level of marginal distributions, they can accumulate during long-term autoregressive prediction and contribute to a gradual loss of dynamical richness when the C-Prior is incorporated into the Koopman-based predictor.

We further note that achieving this level of performance with a C-Prior requires a substantially larger number of parameters. In our experiments, the VAE-based C-Prior employs on the order of more than 10^5 trainable parameters, compared to approximately 300 parameters in the quantum prior. For completeness and fairness, we also tested a VAE constrained to a comparable parameter budget of approximately 300 parameters; in this regime, the model was unable to train stably and collapsed to an overly smooth, mean-field representation, failing to capture meaningful fine-scale statistics. This contrast highlights the parameter efficiency of the Q-Prior in representing multiscale statistical features relevant for chaotic dynamics. A summary of parameter counts for all models considered is provided in Table S3.

S7.6 Integration of the Traditional Model and the Quantum Prior Guidance

In our QIML framework, the integration of quantum and traditional components is achieved by embedding a quantum-learned invariant distribution into the loss function of a traditional machine

learning model tasked with forecasting high-dimensional dynamical systems. This coupling is designed to address a fundamental limitation in traditional neural PDE solvers: while they can capture short-term evolution, their predictive accuracy typically deteriorates over longer horizons due to the accumulation of numerical error and sensitivity to initial conditions.

The traditional machine learning model, constructed using convolutional or transformer-based architectures, is trained to predict the next-step velocity field $\hat{u}_{t+1}$ from a history of past states $\{u_t, u_{t-1}, \ldots\}$. To enhance robustness in long-term rollouts, we incorporate a distributional Q-Prior $p_\theta(x)$ learned by the quantum generator, which approximates the system's invariant measure.

The initial loss formulation includes a reconstruction term and a Kullback–Leibler divergence between the predicted and quantum-learned distributions:

$$\mathcal{L}_{\text{total}} = \mathcal{L}_{\text{recon}} + \mathcal{L}_{\text{unitary}} + \lambda_{\text{KL}}\mathcal{L}_{\text{KL}}, \tag{S30}$$

where

$$\mathcal{L}_{\text{unitary}} = |K^\top K - I|_F^2, \text{ for details, see the main text}, \tag{S31}$$

$$\mathcal{L}_{\text{recon}} = |\hat{u}_{t+1} - u_{t+1}|^2, \tag{S32}$$

and

$$\mathcal{L}_{\text{KL}} = D_{\text{KL}}\left(\hat{q}(x) | p_\theta(x)\right), \tag{S33}$$

with $\hat{q}(x)$ denoting the empirical distribution derived from the predicted field $\hat{u}_{t+1}$.

While this formulation successfully captures the global distributional shape, it often converges to a low-variance mean-field solution, insufficient to preserve local or high-frequency structures critical for short-term dynamics. To mitigate this, we introduce two additional constraints.

First, we add a MMD term to better match higher-order statistics:

$$\mathcal{L}_{\text{MMD}} = |\mathbb{E}x \sim \hat{q}(x)[\phi(x)] - \mathbb{E}x \sim p_\theta(x)[\phi(x)]|_{\mathcal{H}}^2, \tag{S34}$$

where $\phi(x)$ is a feature mapping into a reproducing kernel Hilbert space.

Second, in some cases (TCF), we also incorporate a peak loss to explicitly align dominant modes of the predicted and target distributions:

$$\mathcal{L}_{\text{peak}} = |\text{TopK}(\hat{q}(x)) - \text{TopK}(p_\theta(x))|^2 , \tag{S35}$$

where TopK selects the highest-probability bins from both histograms.

The final training loss is a weighted combination of all four components:

$$\mathcal{L}_{\text{total}} = \mathcal{L}_{\text{recon}} + \lambda_{\text{KL}}\mathcal{L}_{\text{KL}} + \lambda_{\text{MMD}}\mathcal{L}_{\text{MMD}} + \lambda_{\text{peak}}\mathcal{L}_{\text{peak}}. \tag{S36}$$

This composite objective ensures that predictions remain consistent with the invariant statistics of the underlying physical system, while also preserving critical local features necessary for short- and mid-term forecasting. Our results demonstrate that Q-Priors can significantly enhance the stability and accuracy of machine learning models in high-dimensional chaotic regimes.

Fig. S8 presents the spatial distribution of the normalized velocity error across the domain under three modelling regimes. The top panel displays error patterns obtained without Q-Priors, revealing widespread, spatially incoherent deviations with peak errors exceeding 10%. Incorporating a Q-Prior from a classical emulator (middle panel) leads to a pronounced reduction in error magnitude and spatial variance, with the majority of regions falling below 4%. Further improvement is observed when employing the Q-Prior trained on real quantum hardware (bottom panel), where error distributions are both quantitatively reduced and exhibit greater spatial smoothness, indicating enhanced generalization and stability in chaotic regime reconstruction. Fig. S9 shows the TCF velocity magnitude distribution $\hat{q}(x)$ predicted under the same three learning regimes (no Q-Prior, emulator-based Q-Prior, and hardware-based Q-Prior), compared to the empirical target distribution. Without quantum guidance, the model fails to capture the dominant statistical mode—both the modal peak and its amplitude are significantly misaligned. With simulator-based Q-Prior, the predicted distribution nearly perfectly matches the raw data in both location and shape, confirming the Q-Prior's capacity to encode high-dimensional invariant measures with minimal parameterization. Hardware-based Q-Prior introduces moderate deviations, including a downward shift in peak probability and a small modal displacement; however, the distribution remains substantially closer to the ground truth than the classical result.

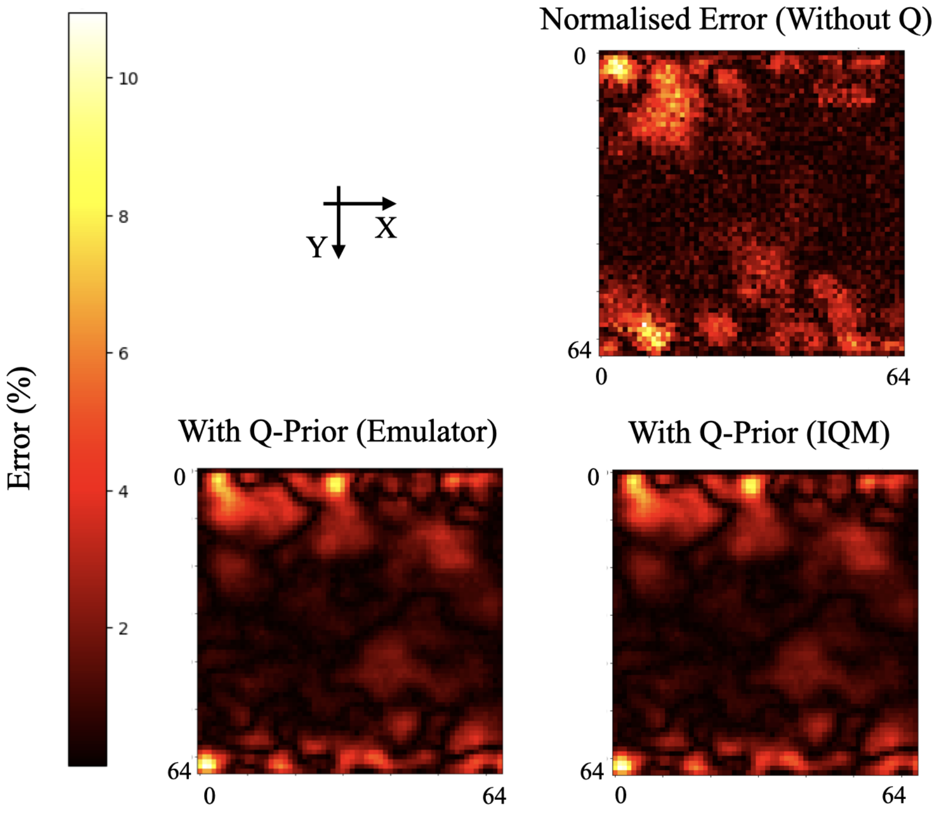

Figure S8: **Diagram of the normalized error of the turbulent channel flow.** The baseline model without the Q-Prior (top right) is compared against the QIML model on a classical emulator (bottom left) and on IQM quantum hardware (bottom right). The QIML model demonstrates a marked reduction in error compared to its classical counterparts.

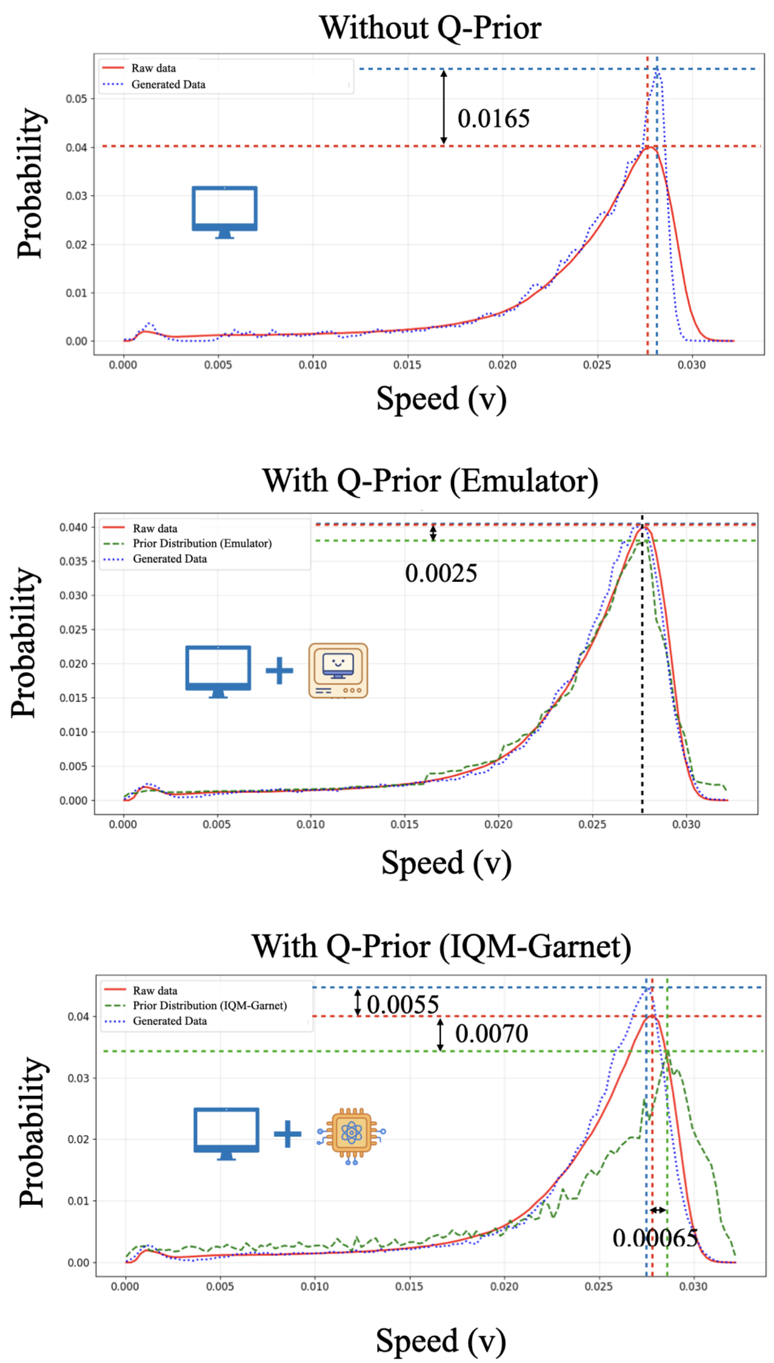

Figure S9: **Diagram of the velocity distribution of the turbulent channel flow predicted under the same three learning regimes (no Q-Prior, emulator-based Q-Prior, and IQM hardware-based Q-Prior), compared to the target distribution.** Without quantum guidance, the model fails to capture the dominant statistical mode—both the modal peak and its amplitude are significantly misaligned. With Q-Prior, the predicted distribution nearly perfectly matches the raw data.

S8 Quantum Parameter Efficiency and Quantum Memory Advantage

This section explains the parameter efficiency and memory advantage for the QIML. According to the parameter numbers in this work, as shown in Table S4, we used 120 parameters on the KS system, 180 on the Kolmogorov flows on the emulator, and 240 or 300 for the TCF system on the hardware and emulator. As is evident, our method uses a remarkably small number of parameters to learn the necessary statistical information. We have already shown the orders-of-magnitude difference in parameter counts compared to classical models in Table S3. Both the parameter efficiency discussed here and the memory advantage that follows originate from the same foundational quantum advantage (*68*): the ability of a quantum circuit to logarithmically compress classical data. This is the fundamental source of the quantum advantage established in our work, a topic we have discussed in detail in section S6. The critical challenge in harnessing this advantage is circumventing the limitations imposed by Holevo's bound. This bound arises because retrieving the complete classical information from the quantum state would necessitate an exponential number of measurements, rendering the approach impractical. This obstacle is overcome because the task of guiding a dynamical model does not necessitate access to the complete classical dataset. Instead, the framework's objective is to extract a statistical summary, the distribution of velocity information, which represents the system's invariant measure. For a chaotic system, such a measure inherently contains far less information than the full dataset from which it is derived. The ability to function with only this compressed distributional information allows the method to bypass the need for an exponential number of measurements. This principle is the foundation for the quantum memory advantage demonstrated empirically for the three systems investigated in this section. Before detailing these quantitative results, we first proceed with a formal theoretical argument for the aforementioned quantum advantage.

S8.1 Theoretical argument for the quantum memory advantage via QIML

Our QIML framework employs a Parameterized Quantum Circuit (PQC), denoted $U(\theta)$, acting on n_q qubits to learn the invariant measure $\mu(x)$ associated with a high-dimensional chaotic dynamical

system. The quantum state generated by the circuit, $|\psi_\theta\rangle = U(\theta)\,|0\rangle^{\otimes n_q}$, resides in a Hilbert space $\mathcal{H}$ of dimension $N = 2^{n_q}$. A naive reconstruction of $|\psi_\theta\rangle$ would indeed require resources that scale exponentially with n_q, apparently contradicting our assertion of efficiency based on a polynomial number of measurements, $S = \text{poly}(n_q)$. Furthermore, Holevo's theorem (*48*) limits the classical information obtainable per measurement to n_q bits. We now formalize why our learning task circumvents these exponential barriers.

The essential insight arises from the structure of the target distribution $\mu(x)$ and the nature of the learning objective. For a dissipative chaotic system with a discrete state space $X \subseteq \{0,1\}^{n_q}$, the invariant measure $\mu(x)$ is supported on a lower-dimensional strange attractor $\mathcal{A} \subset X$. The complexity of $\mu(x)$ is therefore governed not by the ambient dimension N, but by quantities such as its information dimension $D_1(\mu)$ (*110*), which for physical systems typically scales sub-exponentially, often polynomially or even remaining constant with respect to n_q. Let $C(\mu,\epsilon)$ denote the minimal information required to specify $\mu(x)$ up to accuracy ϵ. The compressibility of the invariant measure implies

$$C(\mu,\epsilon) \ll O(N) = O(2^{n_q}). \tag{S37}$$

Furthermore, in simple terms, according to statistical learning theory (*53, 111*), the number of samples S' required to estimate the objective or its gradient with accuracy ϵ scales polynomially with the number of parameters and inverse precision:

$$S' = O(\text{poly}(k, 1/\epsilon)). \tag{S38}$$

Since $k = \text{poly}(n_q, L_{\text{circ}})$, the required number of quantum measurements S' scales polynomially with n_q. Holevo's bound does not impose an exponential limitation here, because we are not attempting full quantum state tomography of $|\psi_\theta\rangle$. Instead, the quantum device is used solely to generate classical samples from $p_\theta(x)$, providing stochastic estimates of the loss function for the classical optimizer. In this setting, the quantum circuit acts as an efficient implicit sampler parameterized by θ, whose complexity is dictated by the number of trainable parameters k.

In conclusion, the overall efficiency of QIML arises from two concurrent, complementary factors:

1. The intrinsic compressibility of the target measure $\mu(x)$, expressed in Eq. (S37). This factor represents the compression advantage arising from the exponentially large Hilbert space,

where a complex classical probability distribution can be efficiently extracted and then compactly encoded within a quantum state.

2. The polynomial sample complexity of learning within the parameterized manifold $\mathcal{P}$, as shown in Eq. (S38). This represents the sampling advantage offered by quantum computing, allowing us to efficiently extract effective information from the complex quantum state.

We firstly proceed to quantify the resulting memory advantage achieved for the three specific systems investigated in this work and will establish the theoretical basis for this efficiency in section S10 below.

S8.2 Kuramoto–Sivashinsky Equation

The raw KS data comprise 1200 trajectories, each recorded for 2000 time steps on a 512-point spatial grid, totalling $\sim 12.3\,\mathrm{GB}$. For training we down-sample to $N_{\mathrm{traj}} = 1200$ trajectories, each with 256 temporal frames and 128 spatial points, leading to a working tensor of shape $(256, 128)$ per trajectory. With double precision (8 bytes per value) a single trajectory occupies

$$256 \times 128 \times 8 = 2.62 \times 10^5 \text{ bytes} \simeq 0.25 \text{ MB}. \tag{S39}$$

Thus, the full down-sampled dataset requires $0.25\,\mathrm{MB} \times 1200 \approx 300\,\mathrm{MB}$. Each trajectory is paired with an individual quantum generator containing $n = 10$ qubits, $L = 4$ circuit layers and 120 rotation parameters, for a raw weight file of $120 \times 8 = 960$ bytes; including metadata the stored checkpoint is ≈ 1.0 kB. Archiving one checkpoint per trajectory, therefore, costs $1200 \times 1.0\,\mathrm{kB} \approx 1.2\,\mathrm{MB}$. Consequently, the learned Q-Priors reduce storage from $\sim 300\,\mathrm{MB}$ to $\sim 1.2\,\mathrm{MB}$, a compression factor of around 250:1 while still capturing the essential statistics used by the classical machine learning model.

S8.3 2D Kolmogorov Flows

The total dataset comprises 40 trajectories, each containing 320 temporal snapshots on a 256×256 grid. For a single velocity component, one snapshot occupies

$$256^2 \times 8 = 524,288 \text{ bytes} \simeq 0.50 \text{ MB}. \tag{S40}$$

so that one complete trajectory requires 0.50 MB $\times 320 \approx 160$ MB and the full set of 40 trajectories stores ~ 6.4 GB. The corresponding quantum generator employs $n = 10$ qubits, $L = 6$ layers and 180 trainable rotation angles. Saved in double precision, one checkpoint is $180 \times 8 = 1{,}440$ bytes $\simeq$ 1.4 kB. Archiving a separate checkpoint for every snapshot therefore costs

$$1.4 \text{ kB} \times 320 = 448 \text{ kB} \simeq 0.44 \text{ MB}. \tag{S41}$$

yielding a compression factor of

$$\frac{160 \text{ MB}}{0.44 \text{ MB}} \approx 3.6 \times 10^2. \tag{S42}$$

while faithfully reproducing the empirical invariant measure.

S8.4 Turbulent Channel Flow

For the TCF benchmark, each snapshot is stored as a 192×192 array of double-precision values (8 bytes per entry). A single velocity component therefore, occupies

$$192^2 \times 8 = 294,912 \text{ bytes} \simeq 0.29 \text{ MB}. \tag{S43}$$

so that the full sequence of 595 snapshots amounts to

$$0.29 \text{ MB} \times 595 \approx 170 \text{ MB}. \tag{S44}$$

Retaining all three velocity components raises the per-trajectory footprint to roughly 500 MB.

The quantum generators used to reproduce the same invariant statistics each employ $n = 15$ qubits, $L = 6$ layers, and 240 rotation angles. Each saved quantum generator checkpoint, with minimal metadata, is about 4.3 kB. Given that 595 such quantum generator models are retained (one for each snapshot), the total storage for the entire set of quantum generator checkpoints amounts to $595 \times 4.3\,\text{kB} \approx 2.5\,\text{MB}$. Relative to the raw three-component trajectory ($\sim 500\,\text{MB}$), this represents a compression factor of approximately 200, which is over two orders of magnitude, while still capturing the statistics required by the classical Koopman machine learning model.

S9 Performance and Statistical Metrics

This section provides the mathematical definitions for the primary metrics used in this paper to evaluate model performance and analyse the system's statistical properties.

S9.1 Temporal Autocorrelation

The temporal autocorrelation is used to measure the memory of the dynamical system, quantifying the correlation of a time series with a delayed version of itself. For a discrete time series u_i (the value at a specific spatial point at time step i), with mean $\bar{u}$ and total length N, the normalized temporal autocorrelation $C(k)$ at a time lag of k steps is defined as:

$$C(k) = \frac{\sum_{i=1}^{N-k}(u_i - \bar{u})(u_{i+k} - \bar{u})}{\sum_{i=1}^{N}(u_i - \bar{u})^2}. \tag{S45}$$

S9.2 Error Metrics

To quantify the difference between the ground truth data and the model predictions, the following error metrics are used.

The absolute error provides a direct, point-wise measure of the deviation between a predicted field $\hat{u}(\mathbf{x})$ and the ground truth field $u(\mathbf{x})$:

$$E_{\text{abs}}(\mathbf{x}) = |u(\mathbf{x}) - \hat{u}(\mathbf{x})|\,. \tag{S46}$$

To specifically quantify how well the model reproduces the temporal correlation structure, we define the normalized relative autocorrelation error E_r. This measures the relative L2 error between the true autocorrelation function $C_{\text{true}}(k)$ and the predicted one $C_{\text{pred}}(k)$:

$$E_r = \frac{||C_{\text{true}}(k) - C_{\text{pred}}(k)||_2}{||C_{\text{true}}(k)||_2}. \tag{S47}$$

S10 Argument for the Quantum Advantage in Representing Chaotic Measures

In section S8.1 and the main text, we identified two theoretical sources of efficiency within the QIML framework: the compressibility of the invariant measure into a polynomial number of quantum parameters, and the polynomial sample complexity associated with learning this representation. In this section, we formalize these arguments using concepts from dynamical systems theory and computational complexity. We argue that the invariant measure of a high-dimensional chaotic

system admits an efficient representation by a PQC, whereas classical generative models generically require super-polynomial resources to achieve comparable accuracy.

Our derivation establishes a logical progression from the physical properties of chaotic dynamics to the geometric structure of the invariant measure, and subsequently to the computational complexity of probabilistic modelling.

Consider a dissipative chaotic dynamical system governed by a map $\Phi : \mathcal{X} \to \mathcal{X}$ on a compact phase space $\mathcal{X} \subset \mathbb{R}^N$. The long-time behaviour is characterized by an invariant probability measure μ, satisfying $\mu(E) = \mu(\Phi^{-1}(E))$ for any measurable set E. Classical ergodic theory guarantees the existence of such invariant measures (*77, 78*), and for high-dimensional chaotic flows such as the KS equation or Navier–Stokes turbulence, μ is typically a Sinai–Ruelle–Bowen (SRB) measure (*112*). For computational purposes, we consider a coarse-graining and discretization procedure mapping the phase space to bit-strings $x \in \{0, 1\}^n$ with $n = O(N)$. The statements below refer to this discretized representation. At this resolution, chaotic invariant measures display several geometric and statistical features that make them difficult to approximate using standard classical generative models.

One key property is that $\mathrm{supp}(\mu)$ forms a strange attractor with non-integer (often fractal) dimension (*113*). The combination of nonlinear stretching and dissipative contraction yields a measure that is singular with respect to the ambient Lebesgue measure. Representing such a measure requires specifying an extensive collection of constraints that rule out exponentially many dynamically forbidden regions of phase space. A second feature is the effective non-Markovian nature of the symbolic dynamics. While correlation functions decay, the conditional distribution of the next state does not collapse to any finite-order Markov approximation at physically relevant resolution. Capturing the statistical structure of μ therefore requires access to information distributed across multiple scales of the attractor. A third feature arises from the Koopman description of the dynamics (*114*). The observable f evolves as $f \circ \Phi^t$, and due to sensitivity to initial conditions, its support typically spreads across all degrees of freedom. This spreading is analogous to operator growth or scrambling in quantum many-body systems. As a result, the invariant measure involves long-range and high-order correlations that do not factorize over low-order interaction graphs.

These properties motivate examining the expressivity limitations of classical generative models. Architectures such as finite-range Markov Random Fields, Restricted Boltzmann Machines with

bounded connectivity, and convolutional networks impose local factorization constraints tied to bounded-treewidth graphs. Recent results on contextual and non-local probability distributions (*32*) suggest that such local models may require super-polynomially many parameters to approximate distributions with global correlation structure. Although a fully rigorous lower bound for SRB measures remains an open problem, the combination of fractal geometry, non-Markovianity, and non-local structure indicates a significant mismatch between chaotic invariant measures and the inductive biases of classical local models.

PQCs offer a contrasting representational framework. Quantum circuits can generate global entanglement at depths scaling polylogarithmically in system size, allowing correlations across all qubits to be established without imposing local factorization constraints. Recent work has shown that multifractal and strongly correlated distributions can be generated efficiently by PQCs (*80*), providing evidence that quantum states can encode structures qualitatively similar to those present in chaotic invariant measures. Furthermore, PQCs serve as universal approximators for unitary transformations. Since the Koopman operator is itself a linear (unitary on an appropriate function space) transformation, coarse-grained versions of its spectral structure may be represented within families of quantum circuits. Although this correspondence is heuristic, it suggests a mechanism by which PQCs can encode dynamical invariants using polynomial resources.

Sampling considerations further differentiate classical and quantum settings. Classical sampling from approximations of μ often relies on Markov Chain Monte Carlo methods. The complex, multimodal, and fractal geometry of chaotic attractors is known to create slow mixing and metastability in high dimensional regimes (*115, 116*), making sampling computationally expensive. In contrast, a PQC of depth $O(\text{poly}(N))$ enables direct sampling from its Born distribution at a cost proportional to circuit depth, avoiding the need for Markov mixing altogether. Moreover, gradients of loss functions involving p_θ can be estimated via standard quantum differentiation rules, and recent theoretical work indicates potential advantages in learning distributional properties from quantum-prepared samples (*50*).

Taken together, these considerations provide a conceptual framework for understanding the empirical behaviour observed in the QIML architecture. The invariant measures of chaotic systems possess geometric complexity and global correlation structure that challenge classical generative models based on local interactions or finite-order dependencies. Quantum circuits, through their

ability to generate global entanglement and approximate high-dimensional dynamical structure, offer a plausible and potentially efficient representational basis for these measures. From another perspective, we note an intriguing analogy between our framework and the dynamical systems perspective on classical machine learning, as discussed in recent work on the physical interpretation of neural PDEs (*117*). In that view, the training process can be conceptualized as a discrete dynamical system evolving towards a local attractor that represents the target truth. Extending this analogy to the quantum domain, our QIML training can be seen as driving the quantum state $|\psi(\theta)\rangle$ within the projective Hilbert space towards a specific quantum attractor—a sub-manifold of states whose Born distributions align with the classical invariant measure. Investigating the convergence properties and geometry of these quantum optimisation landscapes through the lens of discrete dynamical systems offers a fertile ground for future theoretical research. Furthermore, we note that related notions of effective dimensionality reduction have also been observed in purely classical uncertainty quantification and physics-informed machine learning. In particular, Edeling *et al.* (*118*) demonstrated that uncertainty arising from thousands of parameters in classical molecular dynamics force fields can, under a kernel-based sensitivity analysis, be reduced to a low-dimensional active subspace. While the resulting reduction does not typically reach the extreme compression levels observed in the present QIML setting, it nonetheless leads to substantial practical gains for uncertainty quantification. These authors also reported closely similar behaviour when using deep active subspace methods (*117*). These classical results provide an important conceptual parallel: despite the apparent high dimensionality of physical models, the uncertainty and statistical variability most relevant for prediction may reside on a significantly lower-dimensional manifold. From this perspective, the Q-Prior in QIML may be viewed as a complementary mechanism for exploiting such effective compressibility, in which a parameterized quantum circuit implicitly encodes a compact statistical representation without requiring explicit kernel construction or sensitivity ranking. This suggests that QIML may also contribute to the broader effort of dimensionality reduction in uncertainty-aware modelling, particularly in regimes where classical methods achieve only partial compression. While establishing a formal complexity-theoretic separation remains an open direction, the theoretical perspective outlined here offers a coherent explanation for the stability, compression, and robustness properties associated with the Q-Prior in our experiments.

Table S1: Representative quantum resource requirements reported for selected quantum algorithms and application settings. The comparison contextualizes the practical scale of quantum resources used in this work, rather than implying direct performance superiority across fundamentally different computational tasks.

Framework	Task type	Qubits	Circuit depth	Shots per eval.	Reported quantum runtime
QIML (this work)	Offline generative prior for turbulence	< 15	< 20	$< 2 \times 10^4$	< 11 h (one-time)
VQE (*99*)	Molecular ground-state energy	10–50	10^2–10^3	10^5–10^7	Days to weeks
QMMM/Embedding (*100*)	Electronic structure embedding	20–50	$> 10^2$	$> 10^5$	Days to weeks
HHL (*46*)	Linear systems solving	$> 10^3$ (logical)	Deep (FTQC)	N/A	Not NISQ-feasible
Random circuit sampling (*101*)	Sampling benchmark	53	~ 20	$\sim 10^6$	Seconds

Table S2: Comparison of Quantum Processor Specifications for Sirius and Garnet.

**Property**	**Sirius**	**Garnet**
Topology	STAR 24	CRYSTAL 20
Qubits	16	20
Pulse-Level Access	Available	Not available
Native Gates	barrier, cz, measure, move, rx	barrier, cz, measure, rx
Max Circuits / Shots	500 / 20000	500 / 20000
Median T1	29.33 μs	38.96 μs
Median T2 (Ramsey)	23.30 μs	7.78 μs
Median T2 (Echo)	30.20 μs	16.95 μs
Median Rotation Gate Fidelity	99.89 %	99.89 %
Median CZ Gate Fidelity	98.27 %	99.29 %
Median Move-Move Fidelity	98.96 %	Not listed

Table S3: Total parameter counts for the compared models.

**Model**	**Total Parameters**
Q-Prior (in the QIML)	<300
C-Prior (VAE comparable)	124,418
C-Prior (VAE fail)	300
Koopman	4,534,872
FNO	35,971,333
MNO	45,823,681

Table S4: Quantum resources and storage compression achieved by the Q-Prior.

**System**	**Qubits**	**Trainable θ parameters**	**Raw data** (full set)	**Q-Prior file** (full set)	**Compression** (ratio)	**Device**
Kuramoto–Sivashinsky	10	~ 120	300 MB	0.25 MB	$\approx 1.2 \times 10^3 : 1$	Emulator
2D Kolmogorov flow	10	~ 180	400 MB	0.40 MB	$\approx 10^3 : 1$	Emulator
Turbulent channel flow	15	~ 300	500 MB	2.3 MB	$\approx 2.2 \times 10^2 : 1$	Emulator
Turbulent channel flow	10	~ 240	500 MB	2.0 MB	$\approx 2.5 \times 10^2 : 1$	IQM-Garnet

Table S5: Matrix definition used in Section S5.6.

1	1	1	1	1	1	1	1	1	1	1	1	1	1	1	1	1	1	1
8	8	-11	8	8	8	-11	8	-11	-30	-11	8	-11	8	8	8	-11	8	8
1	1	-4	1	1	1	-4	1	-4	12	-4	1	-4	1	1	1	-4	1	1
0	-1	0	1	0	-1	0	1	-1	0	1	-1	0	1	0	-1	0	1	0
0	-1	0	1	0	-1	0	1	4	0	-4	-1	0	1	0	-1	0	1	0
-1	0	0	0	1	-1	-1	-1	0	0	0	1	1	1	-1	0	0	0	1
-1	0	0	0	1	-1	4	-1	0	0	0	1	-4	1	-1	0	0	0	1
-1	-1	-1	-1	-1	0	0	0	0	0	0	0	0	0	1	1	1	1	1
-1	-1	4	-1	-1	0	0	0	0	0	0	0	0	0	1	1	-4	1	1
-2	1	-1	1	-2	1	-1	1	2	0	2	1	-1	1	-2	1	-1	1	-2
-2	1	2	1	-2	1	2	1	-4	0	-4	1	2	1	-2	1	2	1	-2
0	-1	-1	-1	0	1	1	1	0	0	0	1	1	1	0	-1	-1	-1	0
0	-1	2	-1	0	1	-2	1	0	0	0	1	-2	1	0	-1	2	-1	0
0	0	0	0	0	1	0	-1	0	0	0	-1	0	1	0	0	0	0	0
1	0	0	0	-1	0	0	0	0	0	0	0	0	0	-1	0	0	0	1
0	1	0	-1	0	0	0	0	0	0	0	0	0	0	0	-1	0	1	0
0	1	0	-1	0	-1	0	1	0	0	0	-1	0	1	0	1	0	-1	0
-1	0	0	0	1	1	0	1	0	0	0	-1	0	-1	-1	0	0	0	1
1	-1	0	-1	1	0	0	0	0	0	0	0	0	0	-1	1	0	1	-1

REFERENCES

1. L. Biferale, G. Boffetta, A. Celani, B. J. Devenish, A. Lanotte, F. Toschi, Multifractal statistics of Lagrangian velocity and acceleration in turbulence. *Phys. Rev. Lett.* **93**, 064502 (2004).

2. V. A. Galaktionov, J. L. Vázquez, *A Stability Technique for Evolution Partial Differential Equations: A Dynamical Systems Approach*, vol. 56 of *Progress in Nonlinear Differential Equations and Their Applications* (Springer Science & Business Media, 2012).

3. Z. Long, Y. Lu, X. Ma, B. Dong, "PDE-Net: Learning pdes from data," in *International Conference on Machine Learning* (PMLR, 2018), pp. 3208–3216.

4. P. V. Coveney, Sharkovskii's theorem and the limits of digital computers for the simulation of chaotic dynamical systems. *J. Comput. Sci.* **83**, 102449 (2024).

5. M. Klöwer, P. V. Coveney, E. A. Paxton, T. N. Palmer, Periodic orbits in chaotic systems simulated at low precision. *Sci. Rep.* **13**, 11410 (2023).

6. B. M. Boghosian, P. V. Coveney, H. Wang, A new pathology in the simulation of chaotic dynamical systems on digital computers. *Adv. Theory Simul.* **2**, 1900125 (2019).

7. P. V. Coveney, S. Wan, *Molecular Dynamics: Probability and Uncertainty* (Oxford Univ. Press, 2025).

8. Y.-C. Chang, X. Wang, J. Wang, Y. Wu, L. Yang, K. Zhu, H. Chen, X. Yi, C. Wang, Y. Wang, W. Ye, Y. Zhang, Y. Chang, P. S. Yu, Q. Yang, X. Xie, A survey on evaluation of large language models. *ACM Trans. Intell. Syst. Technol.* **15**, 1–45 (2024).

9. A. J. Thirunavukarasu, D. S. J. Ting, K. Elangovan, L. Gutierrez, T. F. Tan, D. S. W. Ting, Large language models in medicine. *Nat. Med.* **29**, 1930–1940 (2023).

10. J. Zhang, J. Huang, S. Jin, S. Lu, Vision-language models for vision tasks: A survey. *IEEE Trans. Pattern Anal. Mach. Intell.* **46**, 5625–5644 (2024).

11. K. Zhou, J. Yang, C. C. Loy, Z. Liu, Learning to prompt for vision-language models. *Int. J. Comput. Vis.* **130**, 2337–2348 (2022).

12. I. Price, A. Sanchez-Gonzalez, F. Alet, T. R. Andersson, A. el-Kadi, D. Masters, T. Ewalds, J. Stott, S. Mohamed, P. Battaglia, R. Lam, M. Willson, Probabilistic weather forecasting with machine learning. *Nature* **637**, 84–90 (2025).

13. K. Bi, L. Xie, H. Zhang, X. Chen, X. Gu, Q. Tian, Accurate medium-range global weather forecasting with 3D neural networks. *Nature* **619**, 533–538 (2023).

14. R. Lam, A. Sanchez-Gonzalez, M. Willson, P. Wirnsberger, M. Fortunato, F. Alet, S. Ravuri, T. Ewalds, Z. Eaton-Rosen, W. Hu, A. Merose, S. Hoyer, G. Holland, O. Vinyals, J. Stott, A. Pritzel, S. Mohamed, P. Battaglia, Learning skillful medium-range global weather forecasting. *Science* **382**, 1416–1421 (2023).

15. M. Cavaiola, F. Cassola, D. Sacchetti, F. Ferrari, A. Mazzino, Hybrid AI-enhanced lightning flash prediction in the medium-range forecast horizon. *Nat. Commun.* **15**, 1188 (2024).

16. H. J. Bae, P. Koumoutsakos, Scientific multi-agent reinforcement learning for wall-models of turbulent flows. *Nat. Commun.* **13**, 1443 (2022).

17. X. I. A. Yang, S. Zafar, J.-X. Wang, H. Xiao, Predictive large-eddy-simulation wall modeling via physics-informed neural networks. *Phys. Rev. Fluids* **4**, 034602 (2019).

18. X. Xue, S. Wang, H.-D. Yao, L. Davidson, P. V. Coveney, Physics informed data-driven near-wall modelling for lattice Boltzmann simulation of high Reynolds number turbulent flows. *Commun. Phys.* **7**, 338 (2024).

19. R. Maulik, O. San, J. D. Jacob, C. Crick, Sub-grid scale model classification and blending through deep learning. *J. Fluid Mech.* **870**, 784–812 (2019).

20. A. Pal, Deep learning emulation of subgrid-scale processes in turbulent shear flows. *Geophys. Res. Lett.* **47**, e2020GL087005 (2020).

21. K. Fukami, Y. Nabae, K. Kawai, K. Fukagata, Synthetic turbulent inflow generator using machine learning. *Phys. Rev. Fluids* **4**, 064603 (2019).

22. M. Z. Yousif, L. Yu, H. Lim, Physics-guided deep learning for generating turbulent inflow conditions. *J. Fluid Mech.* **936**, A21 (2022).

23. M. Raissi, P. Perdikaris, G. E. Karniadakis, Physics-informed neural networks: A deep learning framework for solving forward and inverse problems involving nonlinear partial differential equations. *J. Comput. Phys.* **378**, 686–707 (2019).

24. S. Cheng, M. Bocquet, W. Ding, T. S. Finn, R. Fu, J. Fu, Y. Guo, E. Johnson, S. Li, C. Liu, E. N. Moro, J. Pan, M. Piggott, C. Quilodran, P. Sharma, K. Wang, D. Xiao, X. Xue, Y. Zeng, M. Zhang, H. Zhou, K. Zhu, R. Arcucci, Machine learning for modelling unstructured grid data in computational physics: A review. *Inf. Fusion* **123**, 103255 (2025).

25. I. Goodfellow, J. Pouget-Abadie, M. Mirza, B. Xu, D. Warde-Farley, S. Ozair, A. Courville, Y. Bengio, Generative adversarial networks. *Commun. ACM* **63**, 139–144 (2020).

26. L. Lu, P. Jin, G. Pang, Z. Zhang, G. E. Karniadakis, Learning nonlinear operators via DeepONet based on the universal approximation theorem of operators. *Nat. Mach. Intell.* **3**, 218–229 (2021).

27. Z. Li, N. Kovachki, K. Azizzadenesheli, B. Liu, K. Bhattacharya, A. Stuart, A. Anandkumar, Fourier neural operator for parametric partial differential equations. arXiv:2010.08895 [cs.LG] (2020).

28. V. Vanchurin, Toward a theory of machine learning. *Mach. Learn. Sci. Technol.* **2**, 035012 (2021).

29. G. Carleo, I. Cirac, K. Cranmer, L. Daudet, M. Schuld, N. Tishby, L. Vogt-Maranto, L. Zdeborová, Machine learning and the physical sciences. *Rev. Mod. Phys.* **91**, 045002 (2019).

30. Y. Schiff, Z. Y. Wan, J. B. Parker, S. Hoyer, V. Kuleshov, F. Sha, L. Zepeda-Núñez, DySLIM: Dynamics stable learning by invariant measure for chaotic systems. arXiv:2402.04467 [cs.LG] (2024).

31. M. Cerezo, G. Verdon, H.-Y. Huang, L. Cincio, P. J. Coles, Challenges and opportunities in quantum machine learning. *Nat. Comput. Sci.* **2**, 567–576 (2022).

32. X. Gao, E. R. Anschuetz, S.-T. Wang, J. I. Cirac, M. D. Lukin, Enhancing generative models via quantum correlations. *Phys. Rev. X* **12**, 021037 (2022).

33. A. Kandala, A. Mezzacapo, K. Temme, M. Takita, M. Brink, J. M. Chow, J. M. Gambetta, Hardware-efficient variational quantum eigensolver for small molecules and quantum magnets. *Nature* **549**, 242–246 (2017).

34. P. J. O'Malley, R. Babbush, I. D. Kivlichan, J. Romero, J. R. McClean, R. Barends, J. Kelly, P. Roushan, A. Tranter, N. Ding, B. Campbell, Y. Chen, Z. Chen, B. Chiaro, A. Dunsworth, A. G. Fowler, E. Jeffrey, E. Lucero, A. Megrant, J. Y. Mutus, M. Neeley, C. Neill, C. Quintana, D. Sank, A. Vainsencher, J. Wenner, T. C. White, P. V. Coveney, P. J. Love, H. Neven, A. Aspuru-Guzik, J. M. Martinis, Scalable quantum simulation of molecular energies. *Phys. Rev. X* **6**, 031007 (2016).

35. J. Stokes, J. Izaac, N. Killoran, G. Carleo, Quantum natural gradient. *Quantum* **4**, 269 (2020).

36. S. Sanyal, K. Roy, Neuro-Ising: Accelerating large-scale traveling salesman problems via graph neural network guided localized Ising solvers. *IEEE Trans. Comput. Aided Des. Integr. Circuits Syst.* **41**, 5408–5420 (2022).

37. M. Ghazi Vakili, C. Gorgulla, J. Snider, A. K. Nigam, D. Bezrukov, D. Varoli, A. Aliper, D. Polykovsky, K. M. Padmanabha Das, H. Cox III, A. Lyakisheva, A. H. Mansob, Z. Yao, L. Bitar, D. Tahoulas, D. Čerina, E. Radchenko, X. Ding, J. Liu, F. Meng, F. Ren, Y. Cao, I. Stagljar, A. Aspuru-Guzik, A. Zhavoronkov, Quantum-computing-enhanced algorithm unveils potential KRAS inhibitors. *Nat. Biotechnol.* **43**, 1954–1959 (2025).

38. M. Benedetti, E. Lloyd, S. Sack, M. Fiorentini, Parameterized quantum circuits as machine learning models. *Quantum Sci. Technol.* **4**, 043001 (2019).

39. J. Preskill, Quantum computing in the NISQ era and beyond. *Quantum* **2**, 79 (2018).

40. T. Kubař, M. Elstner, Q. Cui, Hybrid quantum mechanical/molecular mechanical methods for studying energy transduction in biomolecular machines. *Annu. Rev. Biophys.* **52**, 525–551 (2023).

41. L. Böselt, M. Thürlemann, S. Riniker, Machine learning in QM/MM molecular dynamics simulations of condensed-phase systems. *J. Chem. Theory Comput.* **17**, 2641–2658 (2021).

42. T. M. Bickley, A. Mingare, T. Weaving, M. Williams de la Bastida, S. Wan, M. Nibbi, P. Seitz, A. Ralli, P. J. Love, M. Chung, M. Hernández Vera, L. Schulz, P. V. Coveney, Extending quantum computing through subspace, embedding and classical molecular dynamics techniques. *Digit. Discov.* **4**, 3427–3444 (2025).

43. H. Liu, A. J. Valocchi, Q. Kang, Three-dimensional lattice Boltzmann model for immiscible two-phase flow simulations. *Phys. Rev. E* **85**, 046309 (2012).

44. C. Sanavio, S. Succi, Lattice Boltzmann–Carleman quantum algorithm and circuit for fluid flows at moderate Reynolds number. *AVS Quantum Sci.* **6**, 023802 (2024).

45. C. Sanavio, R. Scatamacchia, C. De Falco, S. Succi, Three Carleman routes to the quantum simulation of classical fluids. *Phys. Fluids* **36**, 057143 (2024).

46. A. W. Harrow, A. Hassidim, S. Lloyd, Quantum algorithm for linear systems of equations. *Phys. Rev. Lett.* **103**, 150502 (2009).

47. F. Tennie, S. Laizet, S. Lloyd, L. Magri, Quantum computing for nonlinear differential equations and turbulence. *Nat. Rev. Phys.* **7**, 220–230 (2025).

48. A. S. Holevo, Bounds for the quantity of information transmitted by a quantum communication channel. *Probl. Peredachi Inf.* **9**, 3–11 (1973).

49. H.-S. Zhong, H. Wang, Y. H. Deng, M. C. Chen, L. C. Peng, Y. H. Luo, J. Qin, D. Wu, X. Ding, Y. Hu, P. Hu, X. Y. Yang, W. J. Zhang, H. Li, Y. Li, X. Jiang, L. Gan, G. Yang, L. You, Z. Wang, L. Li, N. L. Liu, C. Y. Lu, J. W. Pan, Quantum computational advantage using photons. *Science* **370**, 1460–1463 (2020).

50. H.-Y. Huang, M. Broughton, J. Cotler, S. Chen, J. Li, M. Mohseni, H. Neven, R. Babbush, R. Kueng, J. Preskill, J. R. McClean, Quantum advantage in learning from experiments. *Science* **376**, 1182–1186 (2022).

51. J.-G. Liu, L. Wang, Differentiable learning of quantum circuit Born machines. *Phys. Rev. A* **98**, 062324 (2018).

52. M. Benedetti, D. Garcia-Pintos, O. Perdomo, V. Leyton-Ortega, Y. Nam, A. Perdomo-Ortiz, A generative modeling approach for benchmarking and training shallow quantum circuits. *npj Quantum Inf.* **5**, 45 (2019).

53. A. Gretton, K. M. Borgwardt, M. J. Rasch, B. Schölkopf, A. Smola, A kernel two-sample test. *J. Mach. Learn. Res.* **13**, 723–773 (2012).

54. M. Budišić, R. Mohr, I. Mezić, Applied Koopmanism. *Chaos* **22**, 047510 (2012).

55. I. Mezić, Koopman operator, geometry, and learning of dynamical systems. *Notices Am. Math. Soc.* **68**, 1087–1105 (2021).

56. S. L. Brunton, M. Budišić, E. Kaiser, J. N. Kutz, Modern Koopman theory for dynamical systems. arXiv:2102.12086 [math.DS] (2021).

57. Z. Li, M. Liu-Schiaffini, N. Kovachki, B. Liu, K. Azizzadenesheli, K. Bhattacharya, A. Stuart, A. Anandkumar, Learning dissipative dynamics in chaotic systems. arXiv:2106.06898 [cs.LG] (2021).

58. S. Succi, *The Lattice Boltzmann Equation for Fluid Dynamics and Beyond* (Oxford Univ. Press, 2001).

59. D. Kochkov, J. A. Smith, A. Alieva, Q. Wang, M. P. Brenner, S. Hoyer, Machine learning–accelerated computational fluid dynamics. *Proc. Natl. Acad. Sci. U.S.A.* **118**, e2101784118 (2021).

60. R. D. Moser, J. Kim, N. N. Mansour, Direct numerical simulation of turbulent channel flow up to $Re_\tau = 590$. *Phys. Fluids* **11**, 943–945 (1999).

61. S. Chen, G. D. Doolen, Lattice Boltzmann method for fluid flows. *Annu. Rev. Fluid Mech.* **30**, 329–364 (1998).

62. X. Xue, H.-D. Yao, L. Davidson, Synthetic turbulence generator for lattice Boltzmann method at the interface between RANS and LES. *Phys. Fluids* **34**, 055118 (2022).

63. D. P. Kingma, M. Welling, Auto-encoding variational bayes. arXiv:1312.6114 [stat.ML] (2013).

64. M. McCabe, P. Harrington, S. Subramanian, J. Brown, Towards stability of autoregressive neural operators. arXiv:2306.10619 [cs.LG] (2023).

65. P. Lippe, B. Veeling, P. Perdikaris, R. Turner, J. Brandstetter, PDE-refiner: Achieving accurate long rollouts with neural PDE solvers. *Adv. Neural Inf. Proces. Syst.* **36**, 67398–67433 (2023).

66. A. R. Barron, Universal approximation bounds for superpositions of a sigmoidal function. *IEEE Trans. Inf. Theory* **39**, 930–945 (2002).

67. I. Goodfellow, Y. Bengio, A. Courville, *Deep Learning* (MIT Press, 2016).

68. H.-Y. Huang, S. Choi, J. R. McClean, J. Preskill, The vast world of quantum advantage. arXiv:2508.05720 [quant-ph] (2025).

69. S. Jerbi, C. Gyurik, S. C. Marshall, R. Molteni, V. Dunjko, Shadows of quantum machine learning. *Nat. Commun.* **15**, 5676 (2024).

70. P. W. Shor, Polynomial-time algorithms for prime factorization and discrete logarithms on a quantum computer. *SIAM Rev.* **41**, 303–332 (1999).

71. D. Gilboa, H. Michaeli, D. Soudry, J. McClean, Exponential quantum communication advantage in distributed inference and learning. *Adv. Neural Inf. Proces. Syst.* **37**, 30425–30473 (2024).

72. I. P. Cornfeld, S. V. Fomin, Y. G. Sinai, *Ergodic Theory*, vol. 245 of *Grundlehren der mathematischen Wissenschaften* (Springer Science & Business Media, 2012).

73. M. C. Caro, H. Y. Huang, N. Ezzell, J. Gibbs, A. T. Sornborger, L. Cincio, P. J. Coles, Z. Holmes, Out-of-distribution generalization for learning quantum dynamics. *Nat. Commun.* **14**, 3751 (2023).

74. C. S. Calude, G. Longo, The deluge of spurious correlations in big data. *Found. Sci.* **22**, 595–612 (2017).

75. K. E. Petersen, K. Petersen, *Ergodic Theory* (Cambridge Univ. Press, 1989).

76. X. Cheng, Y. He, Y. Yang, X. Xue, S. Cheng, D. Giles, X. Tang, Y. Hu, Learning chaos in a linear way. arXiv:2503.14702 [nlin.CD] (2025).

77. G. D. Birkhoff, Proof of the ergodic theorem. *Proc. Natl. Acad. Sci. U.S.A.* **17**, 656–660 (1931).

78. J. V. Neumann, Proof of the quasi-ergodic hypothesis. *Proc. Natl. Acad. Sci. U.S.A.* **18**, 70–82 (1932).

79. R. Pascanu, T. Mikolov, Y. Bengio, "On the difficulty of training recurrent neural networks," in *International Conference on Machine Learning* (PMLR, 2013), pp. 1310–1318.

80. M. Wang, J. Jiang, P. V. Coveney, Parameter-efficient quantum anomaly detection method on a superconducting quantum processor. *Phys. Rev. Res.* **7**, 043094 (2025).

81. L. Abdurakhimov, J. Adam, H. Ahmad, O. Ahonen, M. Algaba, G. Alonso, V. Bergholm, R. Beriwal, M. Beuerle, C. Bockstiegel, A. Calzona, C. F. Chan, D. Cucurachi, S. Dahl, R. Davletkaliyev, O. Fedorets, A. G. Frieiro, Z. Gao, J. Guldmyr, A. Guthrie, J. Hassel, H. Heimonen, J. Heinsoo, T. Hiltunen, K. Holland, J. Hotari, H. Hsu, A. Huhtala, E. Hyyppä, A. Hämäläinen, J. Ikonen, S. Inel, D. Janzso, T. Jaakkola, M. Jenei, S. Jolin, K. Juliusson, J. Jussila, S. Khalid, S.-G. Kim, M. Koistinen, R. Kokkoniemi, A. Komlev, C. Ockeloen-Korppi, O. Koskinen, J. Kotilahti, T. Kuisma, V. Kukushkin, K. Kumpulainen, I. Kuronen, J. Kylmälä, N. Lamponen, J. Lamprich, A. Landra, M. Leib, T. Li, P. Liebermann, A. Lintunen, W. Liu, J. Luus, F. Marxer, Arianne Meijer-van de Griend, K. Mitra, J. K. Moqadam, Jakub Mrożek, H. Mäkynen, J. Mäntylä, T. Naaranoja, F. Nappi, J. Niemi, L. Ortega, M. Palma, M.

Papič, M. Partanen, J. Penttilä, A. Plyushch, W. Qiu, A. Rath, K. Repo, T. Riipinen, J. Ritvas, P. F. Romero, J. Ruoho, J. Räbinä, S. Saarinen, I. Sagar, H. Sargsyan, M. Sarsby, N. Savola, M. Savytskyi, V. Selinmaa, P. Smirnov, M. M. Suárez, L. Sundström, S. Słupińska, E. Takala, I. Takmakov, B. Tarasinski, M. Thapa, J. Tiainen, F. Tosto, J. Tuorila, C. Valenzuela, D. Vasey, E. Vehmaanperä, A. Vepsäläinen, A. Vienamo, P. Vesanen, A. Välimaa, J. Wesdorp, N. Wurz, E. Wybo, L. Yang, A. Yurtalan, Technology and performance benchmarks of IQM's 20-qubit quantum computer. arXiv:2408.12433 [quant-ph] (2024).

82. P. D. Nation, H. Kang, N. Sundaresan, J. M. Gambetta, Scalable mitigation of measurement errors on quantum computers. *PRX Quantum* **2**, 040326 (2021).

83. C. Zhu, R. H. Byrd, P. Lu, J. Nocedal, Algorithm 778: L-BFGS-B: Fortran subroutines for large-scale bound-constrained optimization. *ACM Trans. Math. Softw.* **23**, 550–560 (1997).

84. Z. Zhang, "Improved Adam optimizer for deep neural networks," in *2018 IEEE/ACM 26th International Symposium on Quality of Service (IWQoS)* (IEEE, 2018), pp. 1–2.

85. R. G. Regis, Stochastic radial basis function algorithms for large-scale optimization involving expensive black-box objective and constraint functions. *Comput. Oper. Res.* **38**, 837–853 (2011).

86. J. Romero, J. P. Olson, A. Aspuru-Guzik, Quantum autoencoders for efficient compression of quantum data. *Quantum Sci. Technol.* **2**, 045001 (2017).

87. L. Lamata, U. Alvarez-Rodriguez, J. D. Martín-Guerrero, M. Sanz, E. Solano, Quantum autoencoders via quantum adders with genetic algorithms. *Quantum Sci. Technol.* **4**, 014007 (2018).

88. Y. Ding, L. Lamata, M. Sanz, X. Chen, E. Solano, Experimental implementation of a quantum autoencoder via quantum adders. *Adv. Quantum Technol.* **2**, 1800065 (2019).

89. M. Kieferová, N. Wiebe, Tomography and generative training with quantum Boltzmann machines. *Phys. Rev. A* **96**, 062327 (2017).

90. S. Jain, J. Ziauddin, P. Leonchyk, S. Yenkanchi, J. Geraci, Quantum and classical machine learning for the classification of non-small-cell lung cancer patients. *SN Appl. Sci.* **2**, 1088 (2020).

91. P.-L. Dallaire-Demers, N. Killoran, Quantum generative adversarial networks. *Phys. Rev. A* **98**, 012324 (2018).

92. J. Romero, A. Aspuru-Guzik, Variational quantum generators: Generative adversarial quantum machine learning for continuous distributions. *Adv. Quantum Technol.* **4**, 2000003 (2021).

93. J. Zeng, Y. Wu, J.-G. Liu, L. Wang, J. Hu, Learning and inference on generative adversarial quantum circuits. *Phys. Rev. A* **99**, 052306 (2019).

94. P. Rebentrost, M. Mohseni, S. Lloyd, Quantum support vector machine for big data classification. *Phys. Rev. Lett.* **113**, 130503 (2014).

95. R. Mengoni, A. Di Pierro, Kernel methods in quantum machine learning. *Quantum Mach. Intell.* **1**, 65–71 (2019).

96. H.-Y. Huang, M. Broughton, M. Mohseni, R. Babbush, S. Boixo, H. Neven, J. R. McClean, Power of data in quantum machine learning. *Nat. Commun.* **12**, 2631 (2021).

97. J. Carrasquilla, G. Torlai, R. G. Melko, L. Aolita, Reconstructing quantum states with generative models. *Nat. Mach. Intell.* **1**, 155–161 (2019).

98. X. Gao, Z.-Y. Zhang, L.-M. Duan, A quantum machine learning algorithm based on generative models. *Sci. Adv.* **4**, eaat9004 (2018).

99. Y. Cao, J. Romero, J. P. Olson, M. Degroote, P. D. Johnson, M. Kieferová, I. D. Kivlichan, T. Menke, B. Peropadre, N. P. D. Sawaya, S. Sim, L. Veis, A. Aspuru-Guzik, Quantum chemistry in the age of quantum computing. *Chem. Rev.* **119**, 10856–10915 (2019).

100. B. Bauer, S. Bravyi, M. Motta, G. K.-L. Chan, Quantum algorithms for quantum chemistry and quantum materials science. *Chem. Rev.* **120**, 12685–12717 (2020).

101. F. Arute, K. Arya, R. Babbush, D. Bacon, J. C. Bardin, R. Barends, R. Biswas, S. Boixo, F. G. S. L. Brandao, D. A. Buell, B. Burkett, Y. Chen, Z. Chen, B. Chiaro, R. Collins, W. Courtney, A. Dunsworth, E. Farhi, B. Foxen, A. Fowler, C. Gidney, M. Giustina, R. Graff, K. Guerin, S. Habegger, M. P. Harrigan, M. J. Hartmann, A. Ho, M. Hoffmann, T. Huang, T. S. Humble, S. V. Isakov, E. Jeffrey, Z. Jiang, D. Kafri, K. Kechedzhi, J. Kelly, P. V. Klimov, S. Knysh, A. Korotkov, F. Kostritsa, D. Landhuis, M. Lindmark, E. Lucero, D. Lyakh, S. Mandrà, J. R. McClean, M. McEwen, A. Megrant, X. Mi, K. Michielsen, M. Mohseni, J. Mutus, O. Naaman, M. Neeley, C. Neill, M. Y. Niu, E. Ostby, A. Petukhov, J. C. Platt, C. Quintana, E. G. Rieffel, P. Roushan, N. C. Rubin, D. Sank, K. J. Satzinger, V. Smelyanskiy, K. J. Sung, M. D. Trevithick, A. Vainsencher, B. Villalonga, T. White, Z. J. Yao, P. Yeh, A. Zalcman, H. Neven, J. M. Martinis, Quantum supremacy using a programmable superconducting processor. *Nature* **574**, 505–510 (2019).

102. Y. Kuramoto, *Chemical Oscillations, Waves, and Turbulence* (Courier Corporation, 2003).

103. G. I. Sivashinsky, On flame propagation under conditions of stoichiometry. *SIAM J. Appl. Math.* **39**, 67–82 (1980).

104. G. J. Chandler, R. R. Kerswell, Invariant recurrent solutions embedded in a turbulent two-dimensional Kolmogorov flow. *J. Fluid Mech.* **722**, 554–595 (2013).

105. J. Smagorinsky, General circulation experiments with the primitive equations: I. The basic experiment. *Mon. Weather Rev.* **91**, 99–164 (1963).

106. S. Hou, J. Sterling, S. Chen, G. Doolen, A lattice Boltzmann subgrid model for high Reynolds number flows. *Pattern Formation and Lattice Gas Automata*, 151–166 (1995).

107. Y. Koda, F.-S. Lien, The lattice Boltzmann method implemented on the GPU to simulate the turbulent flow over a square cylinder confined in a channel. *Flow Turbul. Combust.* **94**, 495–512 (2015).

108. J. Latt, B. Chopard, O. Malaspinas, M. Deville, A. Michler, Straight velocity boundaries in the lattice Boltzmann method. *Phys. Rev. E* **77**, 056703 (2008).

109. D. d'Humieres, Multiple–relaxation–time lattice Boltzmann models in three dimensions. *Philos. Trans. R. Soc. A Math. Phys. Eng. Sci.* **360**, 437–451 (2002).

110. J. D. Farmer, Information dimension and the probabilistic structure of chaos. *Z. Naturforsch.* **37**, 1304–1326 (1982).

111. V. Vapnik, An overview of statistical learning theory. *IEEE Trans. Neural Netw.* **10**, 988–999 (1999).

112. J.-P. Eckmann, D. Ruelle, Ergodic theory of chaos and strange attractors. *Rev. Mod. Phys.* **57**, 617–656 (1985).

113. J. D. Farmer, E. Ott, J. A. Yorke, The dimension of chaotic attractors. *Physica D* **7**, 153–180 (1983).

114. I. Mezić, Spectral properties of dynamical systems, model reduction and decompositions. *Nonlinear Dyn.* **41**, 309–325 (2005).

115. M. Jerrum, A. Sinclair, "The Markov chain Monte Carlo method: An approach to approximate counting and integration," in *Approximation Algorithms for NP-Hard Problems*, D. S. Hochbaum, Ed. (PWS Publishing, 1996), pp. 482–520.

116. Y. Bengio, G. Mesnil, Y. Dauphin, S. Rifai, "Better mixing via deep representations," in *International Conference on Machine Learning* (PMLR, 2013), pp. 552–560.

117. S. Succi, On the physical interpretation of neural PDEs. *Math. Mech. Complex Syst.* **13**, 275–286 (2025).

118. W. Edeling, M. Vassaux, Y. Yang, S. Wan, S. Guillas, P. V. Coveney, Global ranking of the sensitivity of interaction potential contributions within classical molecular dynamics force fields. *npj Comput. Mater.* **10**, 87 (2024).
